# Beyond Protein Abundance: Proteoform Diversity and Phosphoproteome Remodeling in Maize (*Zea mays* L.) Responding to Drought and Heat Stresses

**DOI:** 10.3390/proteomes14030044

**Published:** 2026-08-27

**Authors:** Jan Bocianowski

**Affiliations:** Department of Mathematical and Statistical Methods, Faculty of Agriculture, Horticulture and Biotechnology, Poznań University of Life Sciences, Wojska Polskiego 28, 60-637 Poznań, Poland; jan.bocianowski@up.poznan.pl; Tel.: +48-61-848-7143

**Keywords:** *Zea mays*, drought stress, heat stress, proteomics, phosphoproteomics, proteoforms, proteome complexity, post-translational modifications, proteogenomics, top-down proteomics, climate resilience

## Abstract

Drought and heat stresses are among the most important environmental constraints limiting maize (*Zea mays* L.) productivity worldwide. Over the past two decades, advances in mass spectrometry-based proteomics have generated extensive datasets describing proteins with altered abundance in maize tissues exposed to water deficit and elevated temperatures. These studies have identified numerous stress-responsive proteins involved in photosynthesis, energy metabolism, antioxidant defense, proteostasis, signaling pathways, and cellular homeostasis. In parallel, phosphoproteomic investigations have revealed extensive remodeling of phosphorylation networks that regulate stress perception, signal transduction, and adaptive responses. However, most available studies remain focused on protein-level observations and provide limited insight into the molecular diversity underlying stress adaptation. This review synthesizes current knowledge on the proteomic and phosphoproteomic responding of maize to drought and heat stresses through the emerging perspective of proteoform biology. We discuss how phosphorylation, oxidative modifications, proteolytic processing, alternative splicing, and genetic variation contribute to proteoform generation and expand the functional complexity of the maize stress proteome. Particular emphasis is placed on the integration of quantitative proteomics, phosphoproteomics, and proteogenomics as complementary approaches for characterizing stress-responsive molecular networks. We further evaluate current methodological limitations, including the predominance of bottom-up workflows, the underrepresentation of combined-stress studies and reproductive tissues, and the limited application of proteoform-resolved analytical strategies. We propose that future advances in maize stress biology will require a transition from protein-centered analyses toward proteoform-centered investigations capable of resolving functionally distinct molecular species. The integration of top-down proteomics, phosphoproteomics, proteogenomics, and systems-level approaches is expected to provide a more comprehensive understanding of stress adaptation mechanisms and facilitate the identification of molecular determinants of climate resilience. Such efforts may ultimately support the development of maize cultivars better adapted to increasingly challenging environmental conditions.

## 1. Introduction

Maize (*Zea mays* L.) is one of the most important cereal crops worldwide and plays a central role in global food, feed, and bioenergy production [1,2]. However, the increasing frequency and intensity of drought and heat events associated with climate change pose a substantial threat to maize productivity and yield stability [3]. These abiotic stresses often occur simultaneously during critical developmental stages, resulting in complex physiological and molecular responding that cannot be fully understood through genomic and transcriptomic analyses alone [4]. Because proteins constitute the primary functional components of cellular processes, proteomics provides a direct means of investigating the molecular mechanisms underlying stress adaptation in maize [5].

Over the past two decades, advances in mass spectrometry-based proteomics have enabled the large-scale characterization of proteins associated with drought and heat stresses responding [6]. Numerous studies have identified proteins with altered abundance in leaves, roots, kernels, and reproductive tissues exposed to water deficit, elevated temperature, or combined stress conditions [7,8,9,10]. These investigations have revealed the involvement of diverse biological processes, including photosynthesis, energy metabolism, reactive oxygen species detoxification, protein folding, signaling pathways, and cellular homeostasis. Nevertheless, many studies have focused primarily on protein-level identification and abundance changes, while comparatively less attention has been paid to the broader complexity of the maize proteome [11,12,13].

An emerging concept in modern proteomics is that biological function is often mediated not simply by proteins as gene products but by distinct proteoforms generated through alternative splicing, sequence variation, proteolytic processing, and post-translational modifications (PTMs) [14,15]. A simple example is a protein that exists in an unphosphorylated form and in a phosphorylated form; although both forms originate from the same protein sequence, phosphorylation can alter their activity, stability, localization, or interactions. Thus, the same protein can occur as distinct molecular species with different functional properties depending on its modification state. Consequently, changes observed in proteomic datasets may reflect alterations in the abundance or distribution of specific proteoforms rather than changes in a single canonical protein species [16]. Among PTMs, protein phosphorylation represents one of the most important regulatory mechanisms governing stress perception, signal transduction, transcriptional control, metabolic reprogramming, and cellular adaptation [17]. Phosphorylation can rapidly generate functionally distinct proteoforms, thereby increasing proteome complexity and enabling plants to respond dynamically to environmental fluctuations. In maize, phosphoproteomic studies have identified extensive remodeling of phosphorylation networks under drought and heat stresses, yet the integration of these findings with broader proteomic observations remains limited [18,19].

Although numerous reviews have summarized individual aspects of drought- or heat-responsive proteins in maize, a comprehensive synthesis connecting proteomic and phosphoproteomic evidence through the framework of proteoform diversity is currently lacking [18,19]. Existing studies differ considerably in experimental design, tissue selection, stress intensity, developmental stage, sample preparation strategies, phosphopeptide enrichment approaches, and mass spectrometry platforms, making direct comparisons challenging [20,21]. Importantly, not all reported proteomic responding are consistent across studies. For example, fructose-bisphosphate aldolase has been reported to increase or decrease under drought, while changes in ATP synthase abundance are also variable. Such discrepancies indicate that these proteins should not be considered universal biomarkers of drought response. Instead, their abundance appears to depend on experimental and biological context. Furthermore, the predominance of bottom-up proteomic workflows often limits the characterization of specific stress-associated proteoforms and their functional relevance [22]. As a result, critical questions remain regarding the extent to which observed changes in protein abundance reflect alterations in distinct proteoforms and how phosphorylation-driven proteome remodeling contributes to stress resilience [23].

This review aims to provide an integrated overview of the proteomic and phosphoproteomic responding of maize tissues to drought and heat stresses, with particular emphasis on the role of proteoforms in shaping stress adaptation mechanisms. Beyond summarizing current knowledge, we critically evaluate methodological limitations in the existing literature, identify major knowledge gaps, and discuss emerging opportunities for next-generation proteomics. We argue that future progress will depend on the integration of quantitative proteomics, phosphoproteomics, top-down proteomics, proteogenomics, and systems biology approaches capable of resolving proteoform-specific respondings. Such advances are expected to improve our understanding of maize stress tolerance mechanisms and facilitate the development of climate-resilient crop varieties.

## 2. Experimental Landscape of Maize Stress Proteomics

The growing interest in understanding maize adaptation to drought and heat stresses has stimulated the rapid development of proteomic and phosphoproteomic investigations [18,19]. Over the last two decades, advances in mass spectrometry (MS)-based technologies have enabled increasingly comprehensive characterization of proteins and post-translational modifications associated with abiotic stress responding [24]. These studies have generated extensive datasets describing changes in protein abundance across diverse maize tissues and developmental stages, providing valuable insights into stress-related metabolic, physiological, and signaling processes [4]. Most proteomic investigations of drought and heat stresses in maize have focused on photosynthetically active tissues, particularly leaves, due to their central role in carbon assimilation and their high sensitivity to environmental perturbations. Root tissues have also received considerable attention because of their direct involvement in water acquisition and stress sensing [25]. In contrast, relatively few studies have examined reproductive organs such as tassels, pollen, silks, developing kernels, or embryo tissues, despite their critical importance for grain yield stability under adverse environmental conditions [26]. This imbalance has resulted in a substantial knowledge gap regarding tissue-specific proteomic responding and the mechanisms that determine reproductive resilience during stress exposure [27].

Importantly, the most recent maize proteomic and phosphoproteomic studies published in 2025–2026 substantially extend the evidence base considered in earlier surveys. Recent work has addressed combined drought and heat stresses, heat-induced phosphoproteome remodeling, and the relationship between protein abundance and phosphorylation under drought. In particular, comparative proteomics under combined drought and heat stresses has provided new evidence for stress-interactive rather than purely additive proteome responding [24], while recent maize heat-stress studies have expanded the characterization of phosphorylation-dependent signaling and ABA-associated regulatory networks [18,19]. Most recently, simultaneous analysis of the proteome and phosphoproteome in maize leaves and silks under drought demonstrated tissue-specific relationships between protein abundance and phosphorylation, providing direct evidence that stress regulation can occur at the proteoform level [24,27].

The majority of maize stress proteomics studies employ bottom-up proteomic workflows, in which proteins are enzymatically digested into peptides prior to liquid chromatography–tandem mass spectrometry (LC-MS/MS) analysis [28]. This approach offers high throughput and broad proteome coverage and has become the standard methodology for quantitative comparisons among stress treatments. Early investigations frequently relied on two-dimensional gel electrophoresis (2-DE) combined with mass spectrometric identification of protein spots. Although modern LC-MS/MS-based workflows provide substantially greater proteome coverage at the level of canonical proteins, 2-DE has continued to offer unique advantages that remain relevant in plant stress proteomics. In particular, the separation of intact protein species according to isoelectric point and molecular mass enables the visualization of protein isoforms and post-translationally modified protein species that correspond to distinct proteoforms [29]. Consequently, gel-based approaches can provide information on proteome heterogeneity that is not readily apparent in peptide-centric bottom-up workflows. Over the past two decades, improvements in immobilized pH gradient (IPG) technology, fluorescent differential gel electrophoresis (2D-DIGE), image analysis software, and protein identification by high-resolution mass spectrometry have substantially enhanced the reproducibility, sensitivity, and analytical performance of gel-based proteomics. Contemporary plant proteomics increasingly relies on complementary use of gel-based and gel-free methodologies rather than considering them as competing approaches [30,31,32]. As shown in Table 1, each proteomic platform provides a distinct balance between proteome coverage, quantitative performance, and proteoform resolution, highlighting the value of methodological complementarity. The introduction of high-resolution mass spectrometers and shotgun proteomics approaches significantly expanded proteome coverage. Contemporary studies increasingly utilize label-free quantification (LFQ), tandem mass tag (TMT), or isobaric tags for relative and absolute quantification (iTRAQ) strategies to compare protein abundance across experimental conditions. These technologies have enabled the identification of thousands of proteins in a single experiment, substantially improving the resolution of stress-induced proteome remodeling. However, variations in sample preparation, protein extraction protocols, instrument platforms, database search strategies, and statistical criteria often complicate comparisons among independent studies. Consequently, differences reported between datasets may reflect methodological variation in addition to biological responding [33].

Phosphoproteomic analyses represent a rapidly expanding area within maize stress research. Because phosphorylated peptides typically occur at low abundance, enrichment procedures are generally required prior to mass spectrometric analysis. Immobilized metal affinity chromatography (IMAC) and titanium dioxide (TiO_2_)-based enrichment remain the most widely used approaches for phosphopeptide isolation. Combined with high-resolution LC-MS/MS platforms, these methods have facilitated the identification of thousands of phosphorylation sites associated with drought and heat stresses signaling networks. Such studies have revealed extensive phosphorylation dynamics affecting protein kinases, transcription factors, membrane transporters, metabolic enzymes, and proteins involved in photosynthesis and stress defense [19]. Despite these technological advances, several methodological limitations continue to constrain the interpretation of maize stress proteomics datasets. Most studies report protein groups inferred from peptide identifications rather than directly characterizing distinct protein species. As a result, biologically relevant variations arising from alternative splicing, sequence polymorphisms, proteolytic processing, or post-translational modifications are frequently masked within aggregated protein-level measurements. This issue is particularly important in maize, which possesses a highly complex genome and extensive genetic diversity that may contribute substantially to proteome heterogeneity among cultivars and breeding lines [5].

Furthermore, many experimental designs focus on single stress factors applied under controlled laboratory or greenhouse conditions. In contrast, field-grown maize plants are commonly exposed to multiple concurrent stresses, including combinations of drought, heat, nutrient limitations, and pathogen pressure. Although recent studies have begun to address combined drought and heat stresses, the available evidence remains limited compared with single-stress experiments and is still insufficient to resolve how proteome and phosphoproteome responding interact under field-relevant stress combinations. For example, Pfunde et al. [10] recently compared maize leaf proteomes under combined drought and heat stresses and identified genotype-dependent proteomic signatures associated with contrasting stress responding. These findings provide an important step toward understanding combined-stress adaptation, but they remain primarily abundance-based and therefore do not resolve whether the observed differences arise from distinct proteoform distributions or phosphorylation states. Similarly, relatively few studies employ time-resolved sampling strategies capable of distinguishing early signaling events from longer-term acclimation responding [34,35]. An additional challenge concerns the characterization of proteome complexity beyond protein abundance measurements. Current bottom-up workflows provide only indirect information about proteoforms, even though post-translational modifications, particularly phosphorylation, are likely to generate functionally distinct molecular entities that contribute directly to stress adaptation. Consequently, the true diversity of stress-responsive proteoforms in maize remains largely unexplored. Recent advances in acquisition strategies, including data-independent acquisition and ion mobility-enabled proteomics, offer opportunities to improve proteome depth, quantitative reproducibility, and peptide discrimination while retaining the scalability of bottom-up workflows. In particular, four-dimensional DIA approaches incorporating ion mobility provide an additional separation dimension that can reduce spectral complexity and improve the detection of low-abundance peptides and post-translationally modified species. These approaches may therefore help bridge the gap between broad protein-level coverage and the more detailed characterization of stress-responsive proteoforms. Emerging approaches such as top-down proteomics, native mass spectrometry, and integrative proteogenomic analyses offer promising opportunities to address this limitation by enabling direct characterization of intact proteoforms and their modification patterns [36,37]. Although top-down proteomics is frequently regarded as the most direct strategy for proteoform characterization, current MS-intensive top-down workflows remain technically constrained by protein size, sample complexity, protein solubility, and dynamic range. In practice, routine characterization is most successful for relatively small proteins and proteoforms, whereas large multidomain plant proteins remain difficult to analyze comprehensively. This size dependence is particularly relevant to maize stress proteomics, where large and structurally complex plant proteins remain difficult to characterize comprehensively using MS-intensive intact-protein workflows. Taken together, the existing experimental landscape of maize stress proteomics has established a strong foundation for understanding molecular responding to drought and heat stresses [38,39]. These methodological differences define the level of biological information that can be extracted from existing maize stress proteomics datasets and should therefore be considered when interpreting apparent similarities and discrepancies across studies.

### 2.1. Methodological Considerations and Proteoform Resolution

The methodological comparison summarized in Table 1 highlights an important distinction between proteome coverage and proteoform resolution. Gel-based approaches retain information on intact protein species, bottom-up LC-MS/MS provides broad peptide-level coverage, phosphoproteomics captures site-specific regulatory information, and MS-intensive top-down proteomics offers the most direct access to intact proteoforms. These approaches should therefore be regarded as complementary rather than hierarchical, with their relative value determined by the biological question and the molecular level being investigated. Importantly, differences in extraction, digestion, enrichment, database construction, and data-processing strategies can influence the proteins and phosphoproteins detected across studies. Consequently, apparent differences between datasets may reflect both biological variation and differences in analytical resolution.

Table 1 illustrates that no single proteomic methodology is currently capable of comprehensively resolving the molecular complexity of maize stress responding. While bottom-up LC-MS/MS has transformed large-scale protein identification and quantitative proteomics, its peptide-centric nature frequently obscures relationships among coexisting proteoforms. Conversely, gel-based proteomics continues to offer unique advantages for visualizing intact protein species despite lower proteome coverage, whereas MS-intensive top-down proteomics provides the greatest potential for direct proteoform characterization but remains technically challenging for complex plant proteomes. Rather than representing competing or sequentially superior technologies, these methodologies should be regarded as complementary analytical platforms. Future maize stress proteomics will benefit most from integrated experimental workflows combining quantitative bottom-up proteomics, phosphoproteomics, proteogenomics, and targeted top-down validation according to the biological question under investigation.

However, the higher proteoform resolution of top-down approaches should not be interpreted as a straightforward replacement for bottom-up proteomics. MS-intensive top-down proteomics requires intact proteins to remain sufficiently soluble, stable, and amenable to ionization and fragmentation, which becomes increasingly difficult as protein size, structural heterogeneity, and sample complexity increase. The resulting analytical space is therefore much more constrained than in peptide-centric workflows. Large multidomain proteins, highly heterogeneous protein populations, and low-abundance species can be particularly difficult to characterize comprehensively, while the dynamic range of complex plant extracts can limit detection of proteoforms present at low abundance. Consequently, although top-down analysis provides information that cannot be reconstructed reliably from independently measured peptides, its practical application is currently better suited to selected proteins or targeted biological questions.

Cross-study synthesis. Across the maize drought proteomics literature, the most reproducible responding involves four interconnected functional modules: photosynthetic adjustment, redox homeostasis, and protein quality control. The repeated detection of decreased abundance of photosynthetic proteins together with increased abundance of antioxidant enzymes and proteostasis-related proteins suggests that drought adaptation involves a coordinated redistribution of cellular resources from carbon assimilation toward maintenance and protection of cellular structures. In contrast, proteins involved in primary metabolism and signaling show considerably greater variability in both the direction and magnitude of abundance changes. This variability is unlikely to reflect biological inconsistency alone because studies differ substantially in genotype, developmental stage, tissue, drought intensity, duration, and analytical workflow. Importantly, the apparent reproducibility of some protein families should therefore not be interpreted as evidence that the same molecular species are changing in every experiment. Instead, recurring protein identifications may represent conserved functional responding implemented through different proteoform populations. This distinction provides an important explanation for why pathway-level convergence can coexist with substantial protein-level heterogeneity across individual studies.

### 2.2. Sources of Variability and Conflicting Evidence Across Studies

Sources of variability and conflicting evidence across studies. Although several functional protein groups are repeatedly associated with drought and heat responding in maize, the direction and magnitude of changes reported for individual proteins are not always consistent across studies. For example, enzymes involved in central metabolism may exhibit either increased or decreased abundance depending on the experimental context, as illustrated by fructose-bisphosphate aldolase in drought-stressed tissues. Similar variability is observed for proteins involved in energy metabolism, for which changes in abundance may differ between tissues, genotypes, or stress regimes. Therefore, recurrent identification of a protein across studies should not automatically be interpreted as evidence for a uniform quantitative response. Rather, the available evidence suggests that some proteins represent context-dependent components of broader adaptive pathways.

Differences in experimental design provide an important biological explanation for apparently conflicting results. Drought intensity and duration can determine whether a protein reflects an early stress-signaling response, an intermediate acclimation phase, or prolonged damage and resource limitation. Similarly, developmental stage and tissue type strongly influence the physiological functions represented in the proteome. For example, responding detected in leaves primarily reflect changes in photosynthesis, carbon metabolism, and redox regulation, whereas root and reproductive tissues are exposed to distinct physiological constraints. Genotype-dependent differences may further modify the direction or magnitude of protein responding, particularly because maize germplasm differs substantially in stress tolerance and genetic background. Consequently, differences between studies should be interpreted in relation to experimental context rather than treated as simple discordance between abundance and phosphorylations. The clearest examples of apparent contradiction therefore arise when protein abundance is interpreted as a proxy for functional state. The maize dehydrin data provide a direct example: phosphorylation changed despite relatively stable total protein abundance, and the relationship between abundance and phosphorylation differed between leaves and silks. This finding contradicts the assumption that a stable protein abundance necessarily reflects a stable functional state. Similarly, phosphorylation-dependent changes in signaling proteins may occur without detectable changes in their total abundance. Conversely, an increase in protein abundance does not establish that the newly accumulated protein is present in the functionally active phosphorylation state. Thus, phosphoproteomic findings do not generally invalidate bottom-up proteomics; rather, they demonstrate that abundance-based conclusions become incomplete when functional regulation occurs primarily at the post-translational level.

Methodological heterogeneity represents an additional source of apparent disagreement. Differences in protein extraction, sample preparation, digestion procedures, peptide fractionation, mass spectrometry platforms, database annotation, and statistical criteria can influence both the depth of proteome coverage and the proteins detected as significantly altered. These effects are particularly important when comparing phosphoproteomic datasets, because differences in phosphopeptide enrichment strategies, including IMAC and TiO_2_-based approaches, may influence the spectrum of phosphorylation sites identified. Moreover, the predominance of bottom-up workflows means that apparently identical protein identifications may represent different underlying proteoform populations, while distinct proteoforms may be collapsed into a single protein-level assignment. Thus, some discrepancies between studies may reflect differences in analytical resolution rather than genuine biological disagreement.

Accordingly, conflicting findings should be evaluated at multiple levels of biological organization. Concordance at the pathway or functional-module level may be more informative than concordance in the abundance of individual proteins. For example, apparently heterogeneous changes in individual metabolic enzymes may nevertheless reflect a common metabolic reprogramming, whereas recurrent identification of a protein does not necessarily indicate an identical molecular response. A more reliable interpretation therefore requires comparison of studies according to genotype, tissue, developmental stage, stress regime, sampling time, analytical workflow, and proteomic resolution. This framework is particularly important for distinguishing genuine biological heterogeneity from methodological variation and for identifying robust stress-responsive processes that remain conserved despite differences among individual studies.

## 3. Drought-Induced Remodeling of the Maize Proteome

Drought is the most extensively studied of the two major stresses considered in this review and provides the clearest evidence of both conserved functional responding and strong context dependence in the maize proteome. Across tissues and developmental stages, drought-responsive proteins are consistently associated with photosynthesis, energy metabolism, osmotic adjustment, stress defense, protein quality control, and signal transduction [40,41,42]. The major protein groups reported across these studies are summarized in Table 2.

Overall, the proteins summarized in Table 2 reveal a remarkable convergence at the level of functional pathways, but considerably less consistency at the level of individual proteins and their direction of abundance change. This distinction is illustrated by several protein families for which the direction of abundance change varies among studies. For example, fructose-bisphosphate aldolase has been reported with both increased and decreased abundance under drought, and ATP synthase subunits likewise show variable responding, depending on stress severity, tissue, genotype, and experimental design. Such differences indicate that pathway-level convergence does not necessarily imply uniform protein-level responding. Accordingly, the strongest cross-study consensus appears to occur at the level of biological processes rather than individual protein abundance, and this distinction should be considered when interpreting apparently conflicting proteomic results. The scale of these responding varies substantially among experimental systems. For example, an iTRAQ-based analysis of maize leaves identified 3063 proteins, of which 362 showed increased or decreased abundance after three days of drought (214 and 148, respectively), while a comparative study of drought-tolerant and drought-sensitive maize inbreds identified 721 differentially abundant proteins across four experimental comparisons [51]. These values should not be interpreted as contradictory estimates of the maize drought-responsive proteome, because the studies differed in genotype, tissue, treatment duration, and comparison design; rather, they illustrate how strongly the apparent scale of the response depends on experimental context. We propose that this discrepancy may reflect differences in proteoform composition rather than inconsistencies in protein abundance alone, emphasizing the need for future studies that directly characterize intact proteoforms using complementary analytical approaches. Despite considerable differences among experimental designs, drought severity, developmental stages, maize genotypes, and analytical platforms, the proteins summarized in Table 2 consistently converge into four major functional modules: photosynthesis, central carbon and energy metabolism, redox homeostasis, and proteostasis. In contrast, signaling proteins display substantially greater variability among independent studies, suggesting that stress adaptation may depend more strongly on genotype-specific proteoform composition than on total protein abundance alone. These differences indicate that cross-study convergence is stronger at the level of functional modules than at the level of individual protein abundance, emphasizing the context dependence of the maize drought proteome.

One of the most prominent consequences of drought stress is the disruption of photosynthetic processes. Numerous proteomic studies have reported altered abundance of proteins associated with photosystem I, photosystem II, light-harvesting complexes, electron transport chains, and carbon fixation pathways [44,45,46]. Proteins involved in the Calvin–Benson cycle, including ribulose-1,5-bisphosphate carboxylase/oxygenase (Rubisco) and enzymes participating in carbon assimilation, frequently exhibit decreased abundance under prolonged water deficit [47]. These changes are generally accompanied by reduced photosynthetic efficiency and impaired biomass accumulation. At the same time, increased abundance of proteins associated with photoprotection and energy dissipation mechanisms has been observed, suggesting an adaptive response aimed at minimizing photooxidative damage. As shown in Table 2, photosynthesis-related proteins, particularly Rubisco subunits, oxygen-evolving enhancer proteins, chlorophyll a/b-binding proteins, and ATP synthase components, are among the most frequently reported drought-responsive proteins in maize tissues, although the direction and magnitude of abundance changes are not uniform across studies. For example, Rubisco-related proteins have been reported to increase in abundance in some drought experiments, whereas other studies report decreased abundance under prolonged water deficit. This apparent discrepancy is likely influenced by differences in drought duration, tissue developmental state, genotype, and the analytical platform used.

Drought-induced alterations are also evident in central carbon and energy metabolism. Enzymes involved in glycolysis, the tricarboxylic acid cycle, and mitochondrial respiration often display changes in abundance that reflect metabolic reprogramming during stress exposure. Increased abundance of selected glycolytic enzymes has been interpreted as a compensatory mechanism supporting cellular energy production when photosynthetic carbon fixation becomes limited. Similarly, proteins involved in ATP generation and mitochondrial maintenance frequently show altered abundance, highlighting the importance of energy homeostasis during drought adaptation [48]. Oxidative stress represents another major consequence of water deficit. Reduced CO_2_ availability and impaired electron transport promote the generation of reactive oxygen species (ROS), resulting in oxidative damage to proteins, lipids, and nucleic acids [40]. Proteomic analyses frequently identify changes in the abundance of antioxidant enzymes, including superoxide dismutases, catalases, ascorbate peroxidases, glutathione S-transferases, and peroxiredoxins. For example, one iTRAQ study identified 207 differentially accumulated protein species among 3676 trusted proteins under drought, including 111 with increased and 96 with decreased abundance, with antioxidant, photosynthetic, and stress-defense proteins among the major functional categories [52]. However, the direction of change is not uniform for all members of these families. For example, recent quantitative proteomics of maize seedlings found that several HSP20 proteins increased under drought, whereas some HSPs decreased, illustrating that even within a stress-responsive protein family, abundance responding may be heterogeneous. Thus, recurrent detection of an antioxidant or chaperone family should not be equated with a uniform abundance response of all family members. Several antioxidant proteins repeatedly identified in maize drought proteomes, including superoxide dismutases, catalases, ascorbate peroxidases, glutathione S-transferases, and peroxiredoxins, are summarized in Table 2. Nevertheless, the functional significance of individual antioxidant proteins may depend not only on their abundance but also on post-translational modifications that influence enzymatic activity, stability, and intracellular localization.

Proteins associated with osmotic adjustment constitute another important category of drought-responsive components. Water deficit frequently promotes increased abundance of late embryogenesis abundant (LEA) proteins, dehydrins, and other hydrophilic proteins that contribute to cellular protection under dehydration conditions. These proteins are thought to stabilize membranes, preserve protein structure, and maintain cellular integrity during water loss. Their accumulation is often accompanied by changes in proteins involved in compatible solute biosynthesis, including pathways associated with proline and soluble sugar metabolism. Together, these responding contribute to maintaining cellular homeostasis during prolonged drought exposure [49,50]. Maintenance of protein quality represents a critical challenge under stress conditions. Drought-induced protein denaturation and oxidative damage stimulate the accumulation of molecular chaperones and components of protein turnover pathways. Increased abundance of heat shock proteins (HSPs), chaperonins, protein disulfide isomerases, and proteasome-related proteins has been reported in numerous maize studies [18,40]. These proteins support protein folding, prevent aggregation, and facilitate the removal of damaged proteins. Their widespread occurrence across drought-responsive proteomes suggests that proteostasis is a fundamental determinant of stress tolerance.

Signal transduction pathways also undergo substantial remodeling during water deficit. Proteomic investigations have identified changes in the abundance of proteins associated with abscisic acid (ABA) signaling, calcium-mediated responding, kinase cascades, and transcriptional regulation. Although phosphoproteomic studies provide a more direct view of signaling events, conventional proteomic analyses indicate that drought adaptation involves coordinated regulation of multiple signaling components. Such observations support the concept that stress responding emerges from interconnected molecular networks rather than isolated pathways [53]. Importantly, substantial variation exists among maize genotypes regarding the magnitude and nature of drought-induced proteomic changes. Comparative studies involving tolerant and susceptible lines frequently reveal distinct patterns of protein abundance associated with antioxidant capacity, osmotic adjustment, photosynthetic maintenance, and stress signaling. These findings suggest that drought tolerance is not determined by a single molecular mechanism but instead results from the coordinated activity of numerous proteins and pathways. The observed variability also highlights the potential value of proteomic biomarkers for identifying stress-resilient germplasm in breeding programs [54,55].

Despite the extensive body of literature describing drought-responsive proteins in maize, several important limitations remain. Most available studies characterize changes at the protein-group level without resolving the specific proteoforms responsible for stress adaptation [36,56,57]. Alternative splicing, proteolytic processing, and post-translational modifications can generate multiple functionally distinct proteoforms from a single gene product, yet these molecular variants are rarely characterized directly. Importantly, many of the drought-associated proteins listed in Table 2 are known or predicted to exist as multiple proteoforms generated through phosphorylation, oxidation, proteolytic processing, or other post-translational modifications, highlighting a substantial but largely unexplored layer of proteome complexity. Consequently, proteins identified as drought-associated may in fact represent heterogeneous populations of proteoforms with different biological activities. A particularly informative maize example is provided by the drought proteomics study of Benešová et al. [40], in which 2-DE revealed two HSP26 isoforms with distinct drought responding that depended on genotype. In contrast, the corresponding iTRAQ analysis did not preserve this isoform-specific behavior and indicated a more uniform increase in HSP26 abundance. This example illustrates that apparently contradictory findings do not necessarily represent biological disagreement: different analytical platforms may collapse distinct protein species into a common protein-level signal. This observation illustrates that protein-level quantification can conceal biologically relevant heterogeneity among molecular species assigned to the same protein family. It also demonstrates that apparently inconsistent responding between proteomic studies may arise not only from biological variation but from differences in the ability of analytical workflows to resolve individual protein species. Recent evidence further demonstrates that this limitation is not restricted to isoform resolution. Charlton et al. [58] simultaneously quantified protein abundance and phosphorylation in maize leaves and silks exposed to drought and showed that the relationship between total protein abundance and phosphorylation differed between tissues. In particular, dehydrin phosphorylation changed independently of total protein abundance in some cases, indicating that protein-level measurements may obscure tissue-specific regulatory states. Together, these findings demonstrate that protein abundance and phosphorylation can vary independently across tissues, indicating that abundance measurements alone may not capture tissue-specific regulatory states. A comparative iTRAQ study of the drought-tolerant YE8112 and drought-sensitive MO17 inbreds illustrates this context dependence quantitatively: 721 differentially abundant proteins were detected across four comparisons, but only five proteins were reported as overlapping between the two inbred lines, whereas 13 and 84 proteins were specific to YE8112 and MO17, respectively [59].

Phosphorylation, oxidation, acetylation, and other post-translational modifications are likely to influence the behavior of many proteins implicated in drought tolerance. Accordingly, abundance changes should be interpreted together with evidence of post-translational regulation where available. One notable trait emerging from the available drought proteomic literature is the frequent recurrence of metabolic protein families across independent studies. However, recurrence at the level of functional categories does not necessarily imply consistent direction or magnitude of abundance changes for individual proteins. The available evidence therefore suggests a hierarchy of reproducibility: functional modules such as photosynthesis, redox homeostasis, and proteostasis are more consistently represented than individual protein responding, while signaling proteins and some metabolic enzymes show greater context dependence. We propose that this discrepancy is unlikely to reflect biological variability alone but may largely result from differences in proteoform resolution among analytical platforms. Although drought-responsive proteins have been consistently reported for more than two decades, remarkably little attention has been paid to determining whether these proteins represent identical molecular entities or distinct stress-induced proteoforms. Consequently, the current literature provides an increasingly comprehensive inventory of protein abundance changes but still offers only limited mechanistic insight into proteoform-specific adaptation. Taken together, the drought literature indicates that the most reproducible response is not a uniform increase or decrease in individual proteins, but coordinated remodeling of photosynthetic, redox, osmotic-protection, and proteostasis modules. The HSP26 example further suggests that protein-level reproducibility may conceal genotype-dependent differences among individual molecular species. Thus, drought adaptation appears to involve both conserved functional responding and context-dependent molecular states, with the latter potentially arising from differences in proteoform composition. Whether this hierarchy is specific to drought or reflects a broader principle of maize abiotic-stress proteomics becomes apparent only when the drought response is compared directly with the corresponding heat-stress datasets discussed below.

## 4. Heat Stress Effects on Protein Abundance and Proteome Organization

The heat-stress proteome shows substantial overlap with the drought response, but the relative contribution of individual functional modules differs markedly. Whereas drought is strongly associated with coordinated changes in water balance, photosynthesis, and osmotic adjustment, heat stress places particularly strong pressure on protein stability, proteostasis, and thermal maintenance of photosynthetic machinery [60,61]. Heat-responsive protein families and their potential contribution to proteoform diversity are summarized in Table 3. Overall, heat-induced changes in photosynthetic proteins indicate that photosynthetic performance is a major target of heat stress in maize, although the direction and magnitude of individual protein responding depend on genotype, developmental stage, temperature regime, and acclimation history.

A direct comparison of maize responding to drought, heat, and their combination illustrates how strongly stress context influences the observed proteomic response. In one iTRAQ-based study, 5238 proteins were identified, of which 135 showed altered abundance under heat, 68 under drought, and 201 under combined stress. Only one protein was common to the drought and heat treatments, whereas 18 proteins were common to all three conditions; in contrast, 15 proteins were specific to heat, 28 to drought, and 59 to the combined treatment [62]. The relatively small overlap between the single-stress conditions, together with the larger response under combined stress, argues against treating drought and heat as interchangeable proteomic perturbations and supports the view that stress-specific molecular states may arise even when broader functional pathways are shared.

Heat stress also substantially affects cellular energy metabolism, including glycolysis, the tricarboxylic acid cycle, mitochondrial respiration, and ATP production. Although heat-responsive proteins are consistently reported across independent studies, their biological interpretation remains constrained because the majority of available datasets originate from bottom-up proteomics. Consequently, whether identical protein names reported by different authors correspond to the same molecular species or to distinct stress-induced proteoforms remains largely unresolved. This limitation represents one of the major conceptual barriers identified in the present review. Comparison with the drought-responsive proteins summarized in Table 2 indicates that heat stress places a particularly strong emphasis on protein structural integrity and proteostasis, whereas drought produces a broader redistribution involving water balance, photosynthesis, redox homeostasis, and carbon and energy metabolism. The frequent recurrence of molecular chaperones, heat shock proteins, antioxidant enzymes, and photosynthetic proteins across independent studies suggests that maintenance of protein homeostasis constitutes a central component of thermotolerance. However, recurrence of these protein classes does not imply uniform responding of individual proteins. For example, the abundance and proteoform distribution of Rubisco activase can differ between maize cultivars during heat acclimation, illustrating that a conserved functional role may be implemented through distinct molecular responding [63]. However, most available datasets remain based on bottom-up proteomics, making it difficult to determine whether identical protein names reported by different studies correspond to identical molecular species or to distinct stress-induced proteoforms. These changes indicate that heat stress alters not only the photosynthetic machinery but also the downstream metabolic systems required to maintain cellular energy balance.

Proteomic studies have consistently demonstrated that heat stress affects proteins associated with photosynthesis, energy metabolism, protein quality control, antioxidant defense, and cellular signaling. Among these responding, alterations in photosynthesis-related proteins are particularly prominent. Elevated temperatures negatively affect photosystem II stability, electron transport efficiency, and carbon fixation capacity. Consequently, proteins associated with light harvesting, oxygen evolution, and carbon assimilation frequently exhibit decreased abundance during prolonged heat exposure. Reductions in the abundance of Rubisco activase have been reported in several studies and are of particular importance because this enzyme plays a critical role in maintaining Rubisco activity under fluctuating environmental conditions. However, the direction of RCA abundance changes is not universal. Stainbrook et al. [63] reported increased RCA abundance during heat acclimation in maize, accompanied by cultivar-dependent changes in RCA isoform and proteoform abundance. Thus, apparently opposing observations for RCA may reflect differences in genotype, temperature regime, acclimation state, sampling time, and the molecular species being measured rather than a simple contradiction in biological response. The decline of photosynthetic proteins is commonly associated with reduced carbon assimilation and diminished biomass accumulation. As shown in Table 3, Rubisco activase, Rubisco subunits, oxygen-evolving enhancer proteins, chlorophyll a/b-binding proteins, and ATP synthase components are among the most consistently identified proteins exhibiting altered abundance during heat stress. Heat stress also induces substantial changes in proteins involved in cellular energy metabolism. Elevated temperatures increase respiratory demand and alter the balance between energy production and consumption. Proteomic analyses frequently identify changes in the abundance of glycolytic enzymes, tricarboxylic acid cycle components, ATP synthase subunits, and mitochondrial proteins. These alterations likely reflect metabolic adjustments aimed at maintaining cellular energy homeostasis under conditions of thermal stress. However, the direction and magnitude of abundance changes often vary according to stress intensity, duration, developmental stage, and genotype, highlighting the dynamic nature of metabolic adaptation. This variability provides an important counterpoint to the apparent convergence of functional categories: proteins participating in the same metabolic pathway may respond in opposite directions under different experimental conditions. An additional insight emerging from the cross-study comparison is that heterogeneity among reported protein responding should not necessarily be regarded as experimental noise. When differences in protein abundance are reproducibly associated with genotype, tissue, developmental stage, or stress intensity, they may indicate context-dependent proteome states rather than inconsistent biology. In particular, genotype-dependent differences in proteoform composition could represent an underexplored molecular basis of stress resilience. Thus, future comparative studies should not only search for universally stress-responsive proteins but also identify context-specific proteoform states associated with contrasting physiological phenotypes. Such an approach could shift the objective of stress proteomics from identifying universal biomarkers toward defining molecular signatures of resilience within specific genetic and environmental contexts.

One of the most frequently observed responding to heat stress is the accumulation of proteins associated with proteostasis. Elevated temperatures destabilize protein structure and increase the risk of protein unfolding, aggregation, and irreversible damage. Consequently, molecular chaperones constitute one of the largest groups of proteins exhibiting increased abundance during heat stress. Members of the heat shock protein families are repeatedly detected across maize heat stress studies, although individual family members and proteoforms may show different abundance trajectories depending on genotype, tissue, and heat regime. These proteins contribute to protein folding, stabilization of partially denatured proteins, prevention of aggregation, and recovery of damaged cellular functions [64]. Their widespread occurrence across tissues and experimental systems highlights the central importance of protein quality control in thermal adaptation. Members of the HSP family constitute one of the largest functional groups represented in Table 3, emphasizing the pivotal role of proteostasis maintenance in maize thermotolerance. In addition to chaperones, heat stress influences the abundance of proteins involved in protein degradation pathways. Components of the ubiquitin–proteasome system frequently exhibit altered abundance, suggesting enhanced turnover of damaged proteins. Proteases, proteasome subunits, and ubiquitination-related proteins contribute to the selective removal of non-functional proteins, thereby supporting cellular homeostasis. Together with molecular chaperones, these systems form an integrated proteostasis network that is essential for maintaining protein functionality under elevated temperatures.

Heat-induced oxidative stress represents another critical factor shaping proteome organization. Increased membrane fluidity and impaired electron transport promote excessive ROS generation, leading to oxidative modifications of proteins and other cellular macromolecules. Consistent with observations from drought studies, antioxidant enzymes such as superoxide dismutases, catalases, ascorbate peroxidases, glutathione S-transferases, and peroxiredoxins frequently display increased abundance in heat-stressed maize tissues [65]. These proteins contribute to the maintenance of redox balance and help limit oxidative damage. However, their biological activity may depend not only on protein abundance but also on specific post-translational modifications that influence catalytic properties and protein–protein interactions. Several antioxidant proteins listed in Table 3, including superoxide dismutases, catalases, ascorbate peroxidases, glutathione S-transferases, and peroxiredoxins, are known targets of oxidative and phosphorylation-dependent modifications that may generate functionally distinct proteoforms. Heat stress also affects proteins associated with cellular signaling and stress perception. Altered abundance has been reported for calcium-binding proteins, kinases, phosphatases, transcriptional regulators, and hormone-responsive proteins. These molecular components participate in signal transduction networks that coordinate downstream physiological and metabolic responding. Although conventional proteomic approaches provide valuable information regarding abundance changes, many signaling events are mediated primarily through post-translational modifications rather than changes in total protein abundance. Consequently, phosphoproteomic analyses are required to achieve a more comprehensive understanding of heat-responsive signaling pathways.

The impact of heat stress on reproductive tissues deserves particular attention because reproductive development is often more sensitive to elevated temperatures than vegetative growth. Proteomic investigations of pollen, anthers, developing kernels, and reproductive meristems have revealed alterations in proteins associated with carbohydrate metabolism, cytoskeletal organization, protein folding, and stress defense. These changes are thought to contribute to reductions in pollen viability, fertilization efficiency, and grain set frequently observed under heat stress conditions. Nevertheless, reproductive tissues remain underrepresented in current proteomic datasets compared with leaves and roots, highlighting an important area for future research. While many heat-responsive proteins have been identified, the organization of the stress proteome extends beyond simple abundance changes. Increasing evidence suggests that elevated temperatures influence the structural and functional diversity of proteins through alternative splicing, proteolytic processing, and post-translational modifications. Phosphorylation, acetylation, ubiquitination, and oxidative modifications can generate distinct proteoforms with unique biochemical properties, subcellular localization patterns, and interaction networks. Consequently, proteins identified as heat-responsive entities may actually comprise heterogeneous populations of proteoforms that differ substantially in function. Notably, many protein families summarized in Table 3 possess documented post-translational modification sites or undergo proteolytic processing, suggesting that a significant fraction of the observed heat-responsive proteome may consist of unresolved proteoform populations. This issue is particularly relevant for molecular chaperones, antioxidant enzymes, and signaling proteins, which are frequently subject to extensive post-translational regulation. For example, phosphorylation-dependent proteoforms of heat shock proteins may differ in their interaction partners and substrate specificity, while oxidative modifications can alter the activity of antioxidant enzymes. Similarly, multiple proteoforms of signaling proteins may coexist within a single tissue, contributing to the fine-tuning of stress adaptation mechanisms. However, because most maize heat stress studies rely on bottom-up proteomic workflows, direct characterization of these proteoforms remains limited [28]. Consequently, the proteoform diversity potentially associated with the major heat-responsive proteins presented in Table 3 remains largely unexplored.

Taken together, heat stress induces profound changes in both protein abundance and proteome organization in maize [66]. The scale of heat-associated proteome remodeling can be substantial. In a maize endoplasmic reticulum/microsomal proteome, 8306 proteins were detected, of which 1675 showed increased and 708 showed decreased abundance under heat stress; in the same experiment, 1780 proteins carried at least one ubiquitination site. Thus, approximately 29% of the detected proteins showed significant abundance changes, while ubiquitination extended the observed regulatory landscape beyond protein abundance [66]. Current evidence indicates that thermal adaptation depends on coordinated responding involving photosynthetic adjustment, metabolic reprogramming, antioxidant defense, proteostasis maintenance, and stress signaling [10]. Nevertheless, many aspects of heat-responsive proteome complexity remain unresolved. In contrast to drought, the heat-stress literature shows a particularly strong convergence at the level of proteostasis, including heat shock proteins, chaperones, and protein turnover pathways. This suggests that thermal stress places a stronger selective pressure on maintenance of protein integrity, whereas drought involves a broader redistribution of resources among water balance, photosynthesis, redox homeostasis, and protein protection. Importantly, the overlap between the two responding indicates that these are not independent stress programs but partially shared adaptive networks with different functional weighting.

At the level of protein abundance, the comparison between drought and heat reveals both shared responding and distinct patterns of functional weighting. Photosynthetic proteins, antioxidant enzymes, and components of protein quality control recur under both stresses, indicating a common requirement to protect photosynthetic and cellular functions from stress-induced damage. However, drought-responsive proteomes show a stronger representation of proteins associated with water balance, osmotic adjustment, and redistribution of carbon and energy resources, whereas heat stress produces a more pronounced and recurrent response of molecular chaperones, heat shock proteins, and protein turnover pathways. Central carbon and energy metabolism is comparatively heterogeneous under both conditions, with the direction of abundance changes depending strongly on stress intensity, duration, tissue, and genotype. Thus, the overlap between drought- and heat-responsive protein families should not be interpreted as evidence that the two stresses generate equivalent proteomic states; rather, they appear to recruit partially shared functional modules with different abundance-level priorities.

Cross-study synthesis. Across both drought and heat stresses, the strongest convergence occurs at the level of functional modules rather than individual protein abundance. Proteostasis shows particularly strong convergence under heat, whereas drought studies show broader remodeling involving photosynthesis, redox homeostasis, water balance, and carbon and energy metabolism. Molecular chaperones, heat shock proteins, antioxidant enzymes, and several photosynthetic proteins recur across independent studies, but their abundance trajectories remain context-dependent. In contrast, metabolic proteins display greater heterogeneity in both stress conditions, suggesting stronger dependence on stress intensity, exposure duration, developmental stage, genotype, and tissue. This pattern indicates that recurrent identification of a protein family does not necessarily imply a conserved abundance response or an identical molecular state. Moreover, the apparent convergence of heat-responsive protein families should not be equated with convergence of individual proteoforms, because most studies use bottom-up workflows that collapse multiple molecular species into common protein-level assignments. Thus, current evidence supports a conserved functional response to heat stress but does not yet establish whether this response is mediated by conserved proteoforms or by different proteoform populations across genotypes and tissues.

### Comparative Synthesis of Drought and Heat Stresses Responding

Drought and heat stresses induce partially overlapping but clearly distinguishable proteomic states in maize. At the level of protein abundance, photosynthetic proteins, antioxidant enzymes, and components of protein quality control recur under both stresses, indicating shared requirements for maintaining cellular function under adverse conditions. However, the relative contribution of individual functional modules differs between the two stressors. Drought-responsive proteomes show a stronger representation of proteins associated with water balance, osmotic adjustment, and redistribution of carbon and energy resources, whereas heat stress produces a more pronounced and recurrent response involving molecular chaperones, heat shock proteins, and protein turnover pathways. Thus, shared protein families should not be interpreted as evidence that drought and heat generate equivalent proteomic states; rather, the two stresses appear to recruit partially overlapping functional modules with different biological weighting.

The distinction becomes even more apparent when drought and heat occur simultaneously. Comparative proteomic analysis of maize leaves identified 5238 proteins, of which 68 showed significant abundance changes under drought, 135 under heat, and 201 under the combined treatment; only one protein was common to the two single-stress conditions, whereas 18 proteins responded under all three conditions [62]. This limited overlap indicates that drought- and heat-responsive abundance changes cannot be treated as interchangeable signatures. Importantly, the larger response observed under combined stress suggests that simultaneous drought and heat may reorganize the proteome in a non-additive manner rather than simply combining the two single-stress responding. Therefore, shared functional modules may represent common adaptive requirements, whereas individual protein responding remain strongly dependent on stress context.

This comparison also provides an important qualification to protein-level convergence. The recurrence of functional categories across drought and heat does not establish that the same molecular proteoforms mediate these responding. Differences in genotype, tissue, developmental stage, stress intensity, and analytical platform may alter both protein abundance and the proteoform composition represented within a protein family. Consequently, drought and heat should be viewed as partially overlapping but molecularly distinct stress states, providing a rationale for comparative proteoform-resolved studies rather than treating either stressor as a generic abiotic-stress condition.

## 5. Phosphoproteome Dynamics Under Drought and Heat Stresses

**Figure 1:**
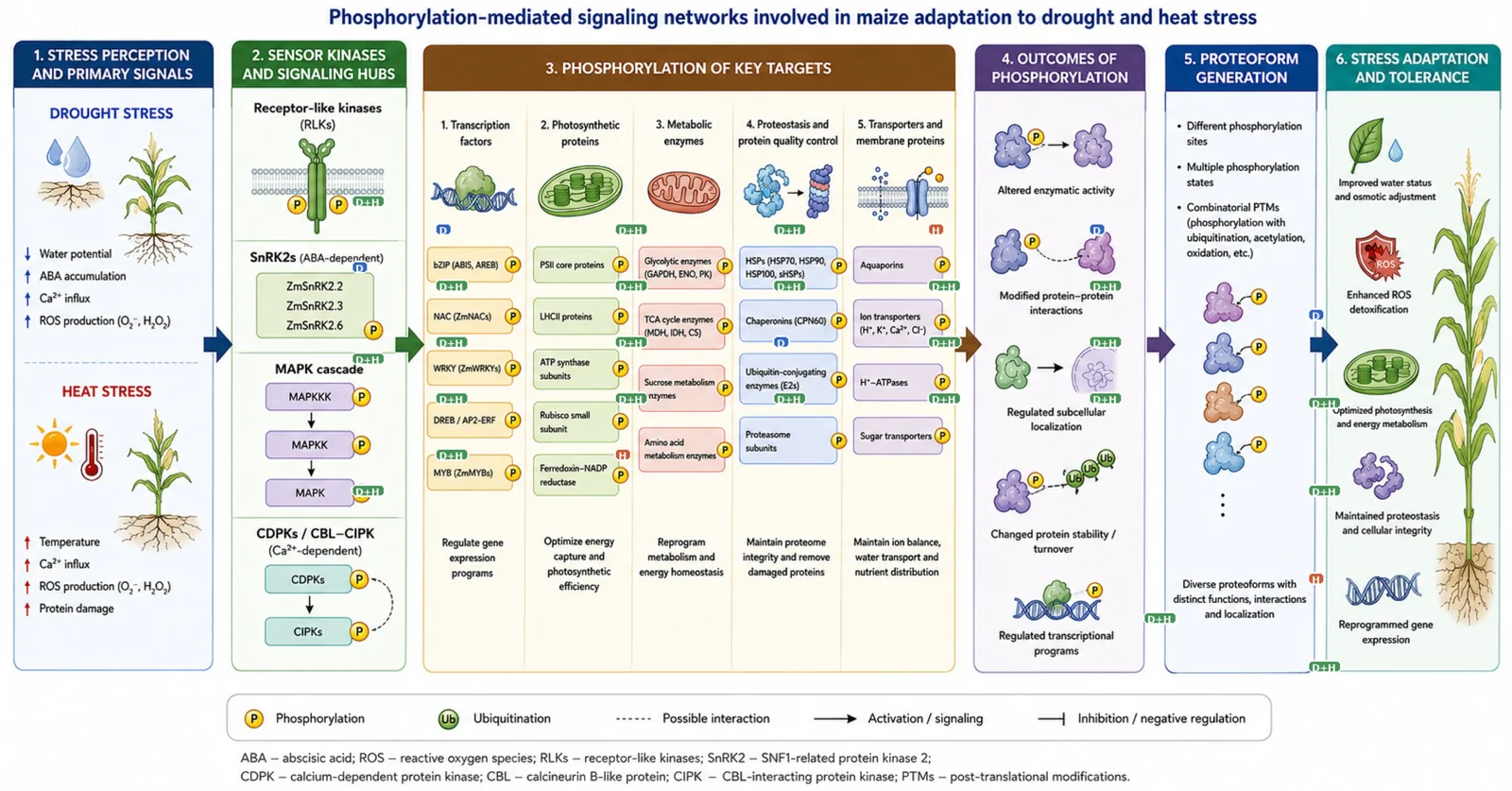
Phosphorylation-mediated signaling networks involved in maize adaptation to drought and heat stresses, highlighting shared and stress-specific responding. D, predominantly drought-associated; H, predominantly heat-associated; D + H, shared between drought and heat; DH, responding specifically associated with combined drought–heat stress. The figure summarizes pathways and protein classes reported in the maize proteomic and phosphoproteomic literature; the assignment of individual nodes reflects the current evidence synthesized in Section 3, Section 4 and Section 5 and Table 2, Table 3 and Table 4. Created in Biorender. Bocianowski. (2026) https://BioRender.com (accessed on 2 July 2026).

One mechanism that may account for rapid changes in protein function without corresponding changes in total abundance is phosphorylation. This is particularly relevant to maize drought and heat responding, in which phosphorylation-dependent signaling networks regulate stress perception, transcriptional reprogramming, metabolism, and protein turnover. Phosphoproteomic studies therefore provide a complementary view of stress adaptation that cannot be obtained from abundance measurements alone. Recent maize studies further strengthen this conclusion. Liu et al. [18] integrated proteomic and phosphoproteomic profiling under heat stress and identified coordinated changes in protein abundance and phosphorylation within ABA-associated signaling networks. Complementary phosphoproteomic analysis by Ma et al. [19] identified 1594 phosphopeptides corresponding to 875 proteins in maize seedlings and highlighted phosphorylation-dependent changes associated with MAPK signaling, protein turnover, translation, and chaperone-related processes. In a large-scale maize proteomic/phosphoproteomic dataset, 14,168 proteins were quantified, whereas 3110 proteins were represented by 8746 phosphorylation sites; only 998 proteins (7.0%) changed significantly in abundance, compared with 26.6% of phosphorylated proteins showing significant changes in phosphorylation level [67]. Together, these studies indicate that heat tolerance is regulated not only through changes in protein abundance but also through rapid remodeling of phosphorylation-dependent regulatory states. Importantly, these findings do not necessarily contradict protein-abundance measurements; rather, they demonstrate that abundance and phosphorylation can change independently, so that an apparently stable protein abundance may coexist with substantial remodeling of its regulatory state. This distinction is critical when interpreting bottom-up datasets, because unchanged abundance does not necessarily imply unchanged protein function. An overview of the major phosphorylation-mediated signaling pathways involved in maize adaptation to drought and heat stresses is presented in Figure 1 [60]. Recent advances in phosphopeptide enrichment strategies and high-resolution mass spectrometry have substantially expanded our understanding of phosphorylation-mediated stress responding. Studies employing immobilized metal affinity chromatography (IMAC), titanium dioxide (TiO_2_)-based enrichment, and tandem mass spectrometry have identified thousands of phosphorylation sites in maize tissues subjected to drought, heat, or combined stress conditions. A representative maize study identified 1367 unique phosphopeptides corresponding to 1039 proteins and 2171 non-redundant phosphorylation sites. Of these, 172 phosphopeptides changed significantly under heat, 149 under drought, and 144 under combined drought–heat stress. Notably, 19 proteins displayed stress-dependent changes at different phosphorylation sites, while 23 phosphopeptides responded specifically to the combined treatment and were not predicted from either single-stress response [68].

**Table 4:** Major classes of phosphorylated proteins identified in maize tissues exposed to drought and heat stresses. The table summarizes representative phosphoproteins reported in phosphoproteomic studies and highlights how phosphorylation contributes to proteoform generation and functional diversification of the maize stress proteome. Because most current datasets are derived from bottom-up phosphoproteomics, many phosphorylation-dependent proteoforms remain incompletely characterized [43].

Phosphorylated Protein Class	Representative Examples	Major Biological Functions	Stress Condition(s)	Potential Contribution to Proteoform Diversity	Representative References
SnRK2 protein kinases	ZmSnRK2.2, ZmSnRK2.3, ZmSnRK2.6	ABA signaling, drought adaptation	Drought	Multiple phosphorylation states regulate kinase activity and substrate specificity	[18,19,68]
MAP kinase cascade components	MAPKKKs, MAPKKs, MAPKs	Signal amplification and stress-response regulation	Drought, Heat	Sequential phosphorylation generates distinct signaling proteoforms	[19,68]
Calcium-dependent protein kinases (CDPKs)	ZmCDPK family proteins	Calcium-mediated signal transduction	Drought, Heat	Site-specific phosphorylation affects downstream target recognition	[19,68]
CBL-interacting protein kinases (CIPKs)	CIPK family proteins	Ion homeostasis and stress signaling	Drought, Heat	Distinct phosphoforms may regulate different transporter systems	[19,68]
Receptor-like kinases (RLKs)	LRR-RLKs, wall-associated kinases	Environmental signal perception	Drought, Heat	Phosphorylation determines receptor activation state	[19,68]
bZIP transcription factors	ABF/AREB proteins	ABA-responsive gene regulation	Drought	Phosphorylation alters DNA binding and transcriptional activity	[18,19,68]
NAC transcription factors	ZmNAC proteins	Abiotic stress adaptation	Drought, Heat	Multiple phosphoforms influence nuclear activity	[19,68]
WRKY transcription factors	ZmWRKY proteins	Stress-responsive transcription	Drought, Heat	Phosphorylation regulates protein stability and target specificity	[19,68]
DREB/AP2-ERF transcription factors	DREB2A-like proteins	Drought- and heat-responsive gene expression	Drought, Heat	Different phosphorylation patterns produce functionally distinct proteoforms	[18,19,68]
Photosystem II proteins	D1, D2, CP43 proteins	Light reactions of photosynthesis	Heat, Drought	Phosphorylation contributes to photosystem repair and acclimation	[19,68]
Light-harvesting complex proteins (LHCII)	LHCB proteins	Energy capture and distribution	Heat, Drought	Dynamic phosphoforms regulate state transitions	[19,68]
ATP synthase subunits	α-, β-subunits	ATP production	Drought, Heat	Phosphorylation influences catalytic efficiency	[19,68]
Rubisco activase	RCA proteins	Maintenance of Rubisco activity	Heat	Phosphorylation may affect thermal stability	[19,68]
Rubisco small subunit	RBCS proteins	Carbon fixation	Drought, Heat	Multiple phosphoforms potentially influence enzymatic regulation	[19,68]
Ferredoxin-NADP reductase	FNR	Electron transport	Heat	Phosphorylation may modify electron transfer efficiency	[19,68]
Glycolytic enzymes	GAPDH, enolase, pyruvate kinase	Energy metabolism	Drought, Heat	Distinct phosphoforms support metabolic reprogramming	[19,68]
TCA cycle enzymes	MDH, IDH, citrate synthase	Cellular respiration	Drought, Heat	Phosphorylation coordinates metabolic flexibility	[19,68]
Sucrose metabolism enzymes	Sucrose synthase, invertases	Carbon allocation	Drought	Multiple phosphoforms may regulate carbon partitioning	[19,68]
Heat shock proteins	HSP70, HSP90, sHSPs	Protein folding and proteostasis	Heat, Drought	Phosphorylation affects chaperone activity and interaction networks	[19,68]
Chaperonins	CPN60 family	Protein quality control	Heat	Distinct phosphoforms may alter substrate affinity	[19,68]
Ubiquitin-related proteins	E2 conjugating enzymes	Protein turnover	Heat, Drought	Crosstalk between phosphorylation and ubiquitination expands proteoform diversity	[19,68]
Proteasome subunits	20S and 26S proteasome proteins	Degradation of damaged proteins	Heat, Drought	Phosphorylation regulates proteasome activity	[19,68]
Aquaporins	PIP1, PIP2 proteins	Water transport	Drought	Phosphorylation directly controls channel opening and trafficking	[19,68]
Ion transporters	H+-ATPases, K+ transporters	Ion homeostasis	Drought, Heat	Phosphorylation modifies transport efficiency and localization	[19,68]
ROS-scavenging enzymes	SOD, APX, CAT, PRX	Antioxidant defense	Drought, Heat	Multiple phosphoforms may differ in catalytic activity	[19,68]
14-3-3 proteins	14-3-3 family proteins	Signaling hub proteins	Drought, Heat	Interact selectively with phosphorylated target proteoforms	[19,68]

An important emerging development is the application of data-independent acquisition (DIA) to phosphoproteomics. DIA-based workflows enable systematic acquisition of fragment ions across predefined mass ranges and can improve reproducibility and quantitative consistency compared with conventional data-dependent acquisition approaches. Plant studies have demonstrated that DIA-based phosphoproteomics can capture rapid phosphorylation changes during environmental stress, including changes that precede measurable alterations in total protein abundance. For maize drought and heat research, DIA-based phosphoproteomics could therefore be particularly valuable for time-resolved and multi-condition experiments in which reproducible quantification of low-abundance phosphopeptides is required. Integration of DIA with ion mobility-enabled acquisition may further increase peptide separation and analytical depth, providing an additional dimension for resolving complex phosphoproteomic datasets.

Phosphorylation-dependent regulation spans several major functional modules, including kinase-mediated signaling, transcriptional regulation, photosynthesis, primary metabolism, protein quality control, and membrane transport. The major classes of phosphorylated proteins identified across maize drought and heat stresses studies are summarized in Table 4 [68]. An important limitation is that evidence derived from single-stress experiments cannot necessarily be extrapolated to combined drought and heat stresses. Because the two stresses share several signaling pathways but impose distinct physiological constraints, their combination may produce interactive rather than additive proteomic responding. The limited number of time-resolved and combined-stress studies therefore prevents firm conclusions about whether responding observed under individual stresses remain conserved under field-relevant stress combinations.

A well-characterized maize example illustrates why phosphorylation should be considered a source of functional proteoform diversity rather than merely a stress-associated phosphosite. The ABA-responsive dehydrin Rab17 (DHN1) exists in phosphorylated and unphosphorylated forms, and phosphorylation of its serine-rich region influences its intracellular distribution and biological activity. Jensen et al. [69] demonstrated that phosphorylation of Rab17 contributes to its nuclear targeting, while subsequent work showed that CK2-mediated phosphorylation is functionally relevant to Rab17 activity under stress conditions [69,70]. Mass-spectrometric mapping further identified multiple phosphorylation sites within the serine-rich region of maize DHN1 [71]. Thus, changes in Rab17 phosphorylation can alter the properties of the protein without necessarily requiring a corresponding change in total protein abundance.

Table 4 highlights that phosphorylation-dependent regulation extends beyond individual signaling proteins and affects transcriptional regulation, photosynthesis, primary metabolism, protein quality control, and membrane transport. A major unresolved issue is that most phosphoproteomic studies identify individual phosphosites without establishing whether multiple phosphorylation events coexist on the same protein molecule. Consequently, future experimental designs should combine site-specific phosphoproteomics with intact-protein measurements and functional validation when the biological question concerns the combined molecular state of a protein. Standardized protocols, time-resolved sampling, and systematic deposition of raw data would further improve cross-study comparability.

One of the earliest events during drought stress is the activation of signaling pathways associated with water-deficit perception. Protein kinases play a pivotal role in these processes, functioning as central regulators of phosphorylation cascades that coordinate downstream adaptive responding. Members of the SNF1-related protein kinase 2 (SnRK2) family are particularly important because they mediate abscisic acid (ABA)-dependent signaling pathways that regulate stomatal closure, osmotic adjustment, and stress-responsive gene expression. Increased phosphorylation of proteins associated with ABA signaling has been reported in multiple phosphoproteomic studies, emphasizing the central role of this pathway in drought adaptation. As illustrated in Figure 1, ABA-dependent SnRK2 signaling operates in parallel with MAPK and calcium-dependent kinase pathways, creating an interconnected regulatory network that coordinates downstream stress responding [18,68]. Mitogen-activated protein kinase (MAPK) cascades constitute another major signaling module influenced by drought and heat stresses. Sequential phosphorylation events involving MAP kinase kinase kinases (MAPKKKs), MAP kinase kinases (MAPKKs), and MAP kinases (MAPKs) facilitate signal amplification and integration across multiple stress-response pathways. Phosphoproteomic analyses indicate that numerous components of MAPK signaling networks exhibit altered phosphorylation status during abiotic stress exposure. These modifications influence downstream transcriptional programs, antioxidant defenses, and cellular metabolic adjustments required for stress acclimation [19].

Calcium-dependent signaling pathways also contribute significantly to phosphoproteome dynamics. Environmental stress frequently triggers transient increases in cytosolic calcium concentrations, which are sensed by calcium-binding proteins and calcium-dependent protein kinases (CDPKs) [68]. Subsequent phosphorylation events regulate diverse cellular targets, including ion transporters, metabolic enzymes, transcription factors, and proteins involved in reactive oxygen species signaling. The widespread detection of phosphorylated calcium-signaling components highlights the importance of calcium as a secondary messenger linking environmental stimuli to adaptive physiological responding. Figure 1 highlights the central position of MAPK and CDPK-mediated phosphorylation cascades in transmitting stress signals from environmental perception to cellular response mechanisms [68]. Representative kinase families and signaling components affected by drought and heat stresses are listed in Table 4 [19]. Phosphorylation-mediated regulation extends beyond signal transduction and directly influences proteins involved in photosynthesis and energy metabolism. Numerous phosphoproteomic studies have reported altered phosphorylation of photosystem components, chlorophyll-binding proteins, ATP synthase subunits, and Calvin–Benson cycle enzymes. These modifications are believed to optimize energy utilization, regulate electron transport, and facilitate acclimation to fluctuating environmental conditions. Representative groups of phosphorylation targets, including photosynthetic proteins, metabolic enzymes, transporters, and transcription factors, are summarized in Figure 1. Importantly, phosphorylation can alter protein function without affecting overall abundance, demonstrating why phosphoproteomic analyses often reveal regulatory mechanisms that remain undetected in conventional proteomic datasets. Several photosynthetic proteins summarized in Table 4 exhibit dynamic phosphorylation patterns that contribute to acclimation under adverse environmental conditions. These findings are not necessarily contradictory because total protein abundance and phosphorylation represent different regulatory layers. Apparent discrepancies between proteomic and phosphoproteomic findings should also be interpreted cautiously. A protein may show unchanged or only modestly altered abundance while undergoing substantial changes in phosphorylation. Conversely, altered protein abundance does not necessarily indicate a corresponding change in activity. Therefore, discordance between abundance-based and phosphorylation-based datasets may represent complementary information rather than contradictory evidence, highlighting the need to integrate multiple molecular layers when interpreting stress responding. Recent maize evidence provides a particularly clear demonstration of this distinction. Charlton et al. [58] quantified total protein and phosphopeptide abundance from the same maize leaf and silk samples under drought stress and found that dehydrin proteoform-related measurements could diverge between tissues. For example, one dehydrin showed coordinated changes in total abundance and phosphorylation in leaves but a different pattern in silks, whereas another dehydrin exhibited altered phosphorylation despite relatively stable total protein abundance. These observations indicate that phosphorylation status cannot be inferred reliably from total protein abundance and provide direct evidence that stress-associated regulation may occur at the level of specific proteoforms rather than protein abundance alone.

Proteins involved in carbohydrate metabolism and mitochondrial function also exhibit extensive phosphorylation dynamics during drought and heat stresses. Glycolytic enzymes, tricarboxylic acid cycle components, and respiratory-chain proteins frequently undergo phosphorylation-dependent regulation, suggesting a role in metabolic reprogramming under stress conditions. Such modifications may enable rapid adjustments in energy allocation and resource utilization when cellular demands change in response to environmental challenges. Another major target of stress-induced phosphorylation is the protein quality control machinery. Heat shock proteins, molecular chaperones, and proteasome-associated proteins frequently display altered phosphorylation status during drought and heat exposure. These modifications may influence substrate recognition, chaperone activity, protein turnover, and interactions with other cellular components. Consequently, phosphorylation contributes not only to signaling processes but also to the maintenance of proteostasis under adverse environmental conditions. Transcriptional regulation represents an additional layer of phosphoproteome remodeling. Numerous transcription factors involved in abiotic stress responding, including members of the bZIP, MYB, NAC, WRKY, and DREB families, are known targets of phosphorylation. Modification of these proteins can alter DNA-binding affinity, protein stability, nuclear localization, and interaction with regulatory cofactors. Through such mechanisms, phosphorylation functions as a molecular bridge connecting stress perception to large-scale transcriptional reprogramming.

Although drought and heat stresses share many phosphorylation-dependent regulatory pathways, important differences also exist. Drought stress is typically characterized by strong activation of ABA-responsive signaling networks and proteins associated with osmotic adaptation, whereas heat stress more prominently affects proteins involved in proteostasis, protein folding, and thermal protection. Nevertheless, substantial overlap occurs between the two stress responding, particularly in signaling pathways involving MAPKs, calcium-dependent kinases, reactive oxygen species signaling, and stress-responsive transcription factors. This overlap suggests the existence of a partially conserved phosphoregulatory network that contributes to adaptation across multiple abiotic stress conditions. From the perspective of proteome complexity, phosphorylation is particularly significant because it generates distinct proteoforms with potentially different biological functions [36]. As depicted in Figure 1, phosphorylation represents a direct mechanism of proteoform generation, linking stress perception and signaling to functional diversification of the maize proteome. A single protein may exist in multiple phosphorylated states, each characterized by unique activity, localization, interaction partners, or stability. The proteoform generation module presented in Figure 1 emphasizes how alternative phosphorylation states can substantially expand proteome complexity without requiring changes in protein abundance. Consequently, phosphoproteomic datasets provide direct evidence that stress adaptation involves the generation of diverse proteoform populations rather than merely changes in protein abundance. For many maize proteins, the number and functional significance of phosphorylation-derived proteoforms remain unknown, indicating that current phosphoproteomic studies likely capture only a fraction of the true regulatory complexity of the stress proteome. Importantly, maize phosphoproteomic data also demonstrate that stress specificity may occur at the level of individual phosphorylation sites. Hu et al. [68] found that 19 proteins were phosphorylated at different sites under drought, heat, and combined drought–heat stress, while a subset of phosphoproteins responded exclusively to the combined treatment. Thus, the same protein can occupy different phosphorylation states depending on the environmental stimulus. This observation provides direct evidence that stress adaptation cannot be fully described by changes in the abundance of canonical proteins and instead requires consideration of stress-specific proteoform states.

The available evidence suggests that phosphorylation is particularly dominant in regulatory pathways requiring rapid changes in protein activity, localization, or interaction rather than changes in total protein abundance. These include ABA-dependent SnRK2 signaling, MAPK cascades, calcium-dependent kinase and CIPK networks, and phosphorylation-dependent regulation of stress-responsive transcription factors. Protein quality-control pathways also appear to contain an important phosphorylation-regulated component, particularly under heat stress. By contrast, changes in photosynthetic capacity, antioxidant capacity, osmotic adjustment, and several metabolic processes are more readily captured through changes in protein abundance. Thus, phosphorylation appears to represent a primary regulatory layer for stress signaling and transcriptional control, whereas abundance measurements more directly reflect longer-term remodeling of metabolic and structural capacity. Despite remarkable advances, several challenges continue to limit phosphoproteomic investigations in maize. Phosphorylation events are often transient and occur at low stoichiometry, complicating their detection and quantification. Differences in phosphopeptide enrichment strategies, mass spectrometry platforms, data processing pipelines, and annotation resources further hinder comparisons among studies. In addition, most available datasets focus on individual stress treatments and single time points, providing only a static view of highly dynamic signaling processes [72]. Future progress will depend on integrating quantitative phosphoproteomics with top-down proteomics, proteogenomics, structural proteomics, and systems biology approaches. Such strategies will facilitate direct characterization of phosphorylation-derived proteoforms and enable a more comprehensive understanding of how phosphorylation shapes stress adaptation at the molecular level. In particular, the combination of phosphoproteomics with proteoform-resolved methodologies offers significant potential for uncovering previously hidden regulatory mechanisms underlying drought and heat tolerance in maize. Overall, current evidence indicates that phosphoproteome remodeling constitutes a central component of maize responding to drought and heat stresses [19]. Through the coordinated action of kinase cascades, calcium-dependent signaling pathways, transcriptional regulators, metabolic enzymes, and protein quality control systems, phosphorylation orchestrates a highly dynamic adaptive network. Deciphering this phosphoregulatory landscape and its associated proteoform diversity will be essential for understanding the molecular basis of maize resilience under increasingly challenging environmental conditions. The phosphoprotein classes presented in Table 4 represent promising targets for future proteoform-resolved investigations aimed at understanding maize stress resilience [68]. These observations advance the interpretation of maize stress proteomics beyond protein abundance: stress specificity may be encoded in the regulatory state of shared proteins rather than in their total abundance. In particular, differential phosphorylation of common protein substrates provides a plausible mechanism through which drought and heat can generate distinct functional responding while engaging overlapping signaling networks.

Taken together, the phosphoproteomic evidence indicates that phosphorylation contributes to stress adaptation through a distributed regulatory network rather than through a single dominant pathway. Kinase-mediated signaling provides the upstream regulatory layer, whereas phosphorylation of transcriptional regulators, photosynthetic and metabolic proteins, protein quality-control components, and membrane transporters represents downstream functional implementation.

## 6. Proteoform Biology and Experimental Evidence in Maize

Direct experimental evidence for proteoform heterogeneity in maize is emerging from studies showing that distinct molecular forms can respond differently to abiotic stress [68,73]. In drought-stressed maize tissues, total dehydrin abundance and phosphorylation were shown to change independently and in a tissue-dependent manner [63]. These observations provide experimental evidence that proteoform composition, rather than protein abundance alone, can contribute to the functional response of maize to abiotic stress. These maize-specific observations further indicate that genotype, tissue, developmental stage, and stress intensity can influence which molecular forms of a protein are represented under stress. This model provides a potential explanation for two recurrent features of the maize stress proteomics literature: the relatively strong convergence of functional pathways across independent studies and the substantially lower reproducibility of individual protein-level responding. Under this interpretation, drought and heat activate a partially shared proteomic backbone, while genotype, tissue, developmental stage, stress intensity, and signaling context determine which proteoforms within these functional modules become predominant. Proteome remodeling should therefore be viewed as a change in the composition and functional state of protein populations rather than solely as a change in the abundance of individual proteins.

This example illustrates a broader limitation of two decades of maize drought proteomics. The field has become highly effective at identifying recurrent drought-responsive protein groups, but much less effective at determining which molecular species within these groups are functionally associated with tolerance. Drought tolerance is a quantitative phenotype emerging from coordinated responding across genotypes, tissues, developmental stages, and time scales, whereas most proteomic studies provide protein-group abundance measurements from a limited biological context. Consequently, repeated identification of the same protein does not establish that the same molecular species, or the same functional state of that protein, is responsible for adaptation. The persistence of this gap therefore reflects not a lack of proteomic coverage, but insufficient resolution of the molecular states that connect protein abundance to physiological tolerance.

Among the mechanisms generating proteoform diversity, phosphorylation is particularly relevant to abiotic stress responding. As discussed in the previous section, drought and heat stresses trigger extensive phosphorylation-dependent signaling networks that affect proteins involved in signal transduction, photosynthesis, metabolism, protein quality control, and transcriptional regulation. Importantly, phosphorylation can generate multiple functionally distinct molecular species from a single protein, creating a diverse population of phosphoproteoforms that differ in activity, stability, and interaction capacity. The conceptual relationship between phosphorylation events and proteoform generation is illustrated in Figure 1. Thus, phosphoproteomic remodeling should be viewed not only as a signaling process but also as a major contributor to proteome complexity during stress adaptation [74]. In addition to phosphorylation, oxidative modifications represent another important source of proteoform diversity under drought and heat stresses. Both stress conditions promote reactive oxygen species accumulation, leading to the oxidation of amino acid residues such as cysteine, methionine, and tyrosine. These modifications may alter enzyme activity, protein stability, or protein–protein interactions and can function either as damaging events or as regulatory signals. Antioxidant enzymes, photosynthetic proteins, and metabolic enzymes are particularly susceptible to oxidative modifications, suggesting that stress-induced oxidative proteoforms may play a previously underappreciated role in maize adaptation [74].

Proteolytic processing also contributes to proteoform generation during environmental stress. Selective cleavage of precursor proteins can produce shorter molecular variants with altered biological functions, subcellular localization, or regulatory properties [75,76]. Heat stress, in particular, is associated with increased protein turnover and activation of proteolytic pathways, potentially expanding the diversity of proteoforms present within stressed tissues. However, because most maize proteomic studies rely on peptide-based protein identification, the characterization of proteolytically generated proteoforms remains limited [77]. Alternative splicing provides an additional mechanism through which proteome complexity can be expanded. Plant genomes frequently generate multiple transcript isoforms from a single gene, particularly under stressful environmental conditions [78,79,80,81]. Several transcriptomic studies have demonstrated extensive alternative splicing in maize exposed to drought and heat stresses, affecting genes involved in signaling, transcriptional regulation, and stress defense [78,79,80]. Although relatively few studies have directly linked alternative splicing events to corresponding protein products, these observations suggest that transcript-derived proteoforms may contribute significantly to stress adaptation. Integrating transcriptomic, proteomic, and proteogenomic datasets will therefore be essential for resolving the biological significance of stress-associated splice variants [81,82]. Nevertheless, proteogenomic evidence alone is generally insufficient to establish the existence of specific intact proteoforms and should ideally be complemented by proteoform-resolved analytical workflows. Several drought-responsive proteins summarized in Table 2, including dehydrins, LEA proteins, antioxidant enzymes, and aquaporins, are likely represented by multiple stress-associated proteoforms. Similarly, many heat-responsive proteins listed in Table 3, particularly heat shock proteins, chaperonins, and proteasome-associated proteins, undergo extensive post-translational regulation that may generate functionally distinct proteoforms.

The importance of proteoforms becomes particularly evident when considering proteins that occupy central positions within stress-response networks. Heat shock proteins, antioxidant enzymes, aquaporins, kinases, transcription factors, and photosynthetic proteins are all known targets of post-translational regulation [56,75]. Consequently, the functional state of these proteins may depend less on total abundance than on the relative distribution of distinct proteoforms [56,75,83]. For example, phosphorylated and non-phosphorylated forms of a transcription factor may differ substantially in DNA-binding activity, while oxidation-sensitive proteoforms of antioxidant enzymes may display different catalytic properties. Similar principles likely apply to many proteins identified in maize drought and heat stresses proteomes [10]. Numerous phosphoprotein classes summarized in Table 4 illustrate how phosphorylation contributes directly to the generation of stress-responsive proteoforms [83,84,85,86].

Based on the current maize evidence, priority targets for proteoform-resolved studies can be organized into three tiers. Tier 1 comprises HSP26 and Rab17/DHN1, for which maize-specific evidence already indicates molecular heterogeneity and/or independent regulation of abundance and phosphorylation. Tier 2 includes HSPs and molecular chaperones, dehydrins/LEA proteins, stress-responsive transcription factors, and key signaling proteins, which are repeatedly associated with drought or heat responding and are subject to multiple regulatory mechanisms. Tier 3 comprises selected photosynthetic and metabolic proteins for which recurrent abundance and phosphorylation changes suggest potentially important, but currently less directly characterized, proteoform diversity.

## 7. Current Gaps and Testable Research Agenda

**Figure 2:**
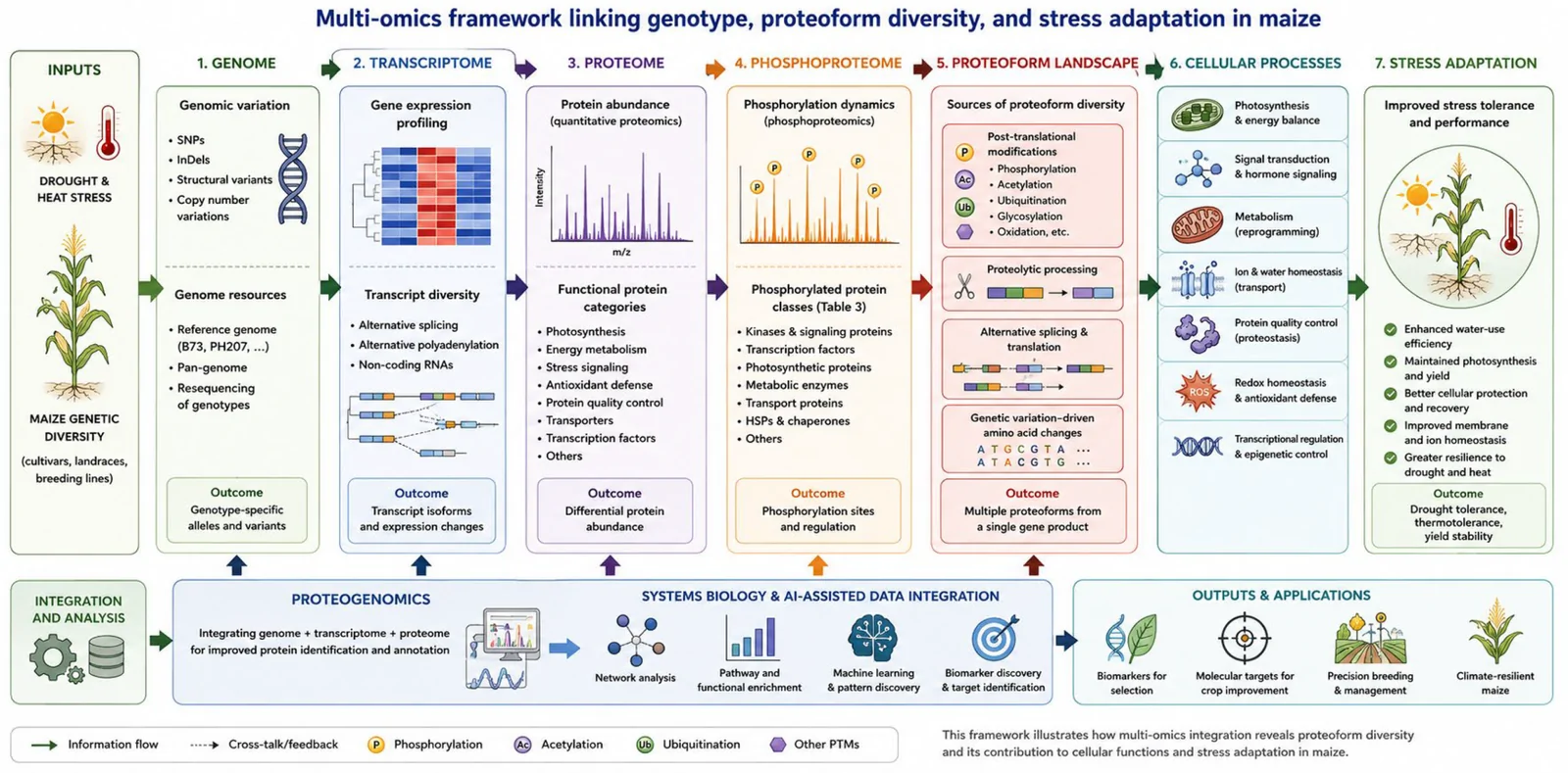
Multi-omics framework linking genotype, proteoform diversity, and stress adaptation in maize. Created in Biorender. Bocianowski. (2026) https://BioRender.com (accessed on 2 July 2026).

The experimental evidence summarized above establishes that proteoform heterogeneity is biologically relevant in maize, but its prevalence, determinants, and relationship to stress tolerance remain poorly defined. Several methodological and biological gaps currently limit the transition from isolated examples to a predictive framework [72]. Proteogenomics addresses these limitations by integrating genomic, transcriptomic, and proteomic information to improve protein identification and characterization. In a typical proteogenomic workflow, genomic and transcriptomic datasets are used to generate customized protein sequence databases that reflect the specific genetic composition of the studied biological material. These databases can then be utilized for mass spectrometry data interpretation, enabling the identification of novel protein variants, alternative translation products, splice isoforms, and genotype-specific proteoforms. Such an approach is particularly valuable in maize because of its extensive genetic diversity and complex genome organization. The proteogenomic integration layer shown in Figure 2 illustrates how genomic and transcriptomic information can improve the identification of genotype-specific proteins and proteoforms [82,86,87].

Importantly, proteogenomics should not be viewed as a direct proteoform characterization strategy. Rather, it improves the interpretation of proteomic datasets by providing customized sequence databases that facilitate the identification of protein variants, alternative splice products, and previously unannotated coding sequences. Consequently, proteogenomics can reveal potential sources of proteoform diversity, but the direct characterization of intact proteoforms generally requires complementary analytical approaches such as gel-based proteomics or top-down proteomics [82,88,89,90,91]. Proteogenomics therefore has a complementary rather than substitutive role in a proteoform-centered workflow. Its principal strength is the improvement of sequence-space coverage by enabling peptide-spectrum matching against genotype- or tissue-specific protein databases. However, identification of peptides corresponding to a sequence variant, splice product, or previously unannotated coding region does not establish how that variant is combined with other sequence features or PTMs within the same intact protein molecule. Proteogenomic analyses may therefore reveal that multiple molecular forms are possible, while remaining unable to determine which combinations coexist as intact proteoforms or which molecular species are functionally relevant. In addition, customized databases increase computational complexity and require careful control of peptide-spectrum matching and false-discovery rates, particularly when large numbers of genotype-specific sequence variants are incorporated. Thus, proteogenomics can substantially improve the identification and interpretation of maize protein diversity, but its evidence should not be equated with direct proteoform characterization (Table 1).

The principal value of top-down proteomics lies in questions that require preservation of molecular connectivity. For example, bottom-up phosphoproteomics can establish that a protein is phosphorylated at several sites, but independent peptide measurements do not necessarily establish whether these modifications coexist on the same protein molecule. Similarly, peptide-level sequence variants and PTMs can be detected separately without demonstrating their co-occurrence within a single proteoform. Intact-protein analysis can therefore address whether specific combinations of phosphorylation, sequence variation, proteolytic processing, or other PTMs define discrete molecular species and whether these species change differentially under drought or heat stress. These questions are particularly relevant for maize proteins that emerge as priority candidates from abundance- and phosphoproteomic datasets. Importantly, resolving a proteoform does not by itself demonstrate functional divergence. Establishing that two proteoforms differ in activity, localization, stability, interaction partners, or contribution to stress tolerance requires complementary biochemical, genetic, and physiological validation.

Emerging analytical technologies offer promising opportunities to address these challenges. Emerging intact-protein approaches provide an essential complement to the discovery-oriented workflows discussed in Section 2. In particular, targeted top-down proteomics can determine whether sequence variants, PTMs, and proteolytic processing events coexist within the same protein molecule and can therefore directly test proteoform-level hypotheses. Given its current technical constraints, this approach is most appropriate for selected proteins identified through quantitative proteomics and phosphoproteomics [24,37,84].

The complementary nature of these approaches is therefore critical. Proteogenomics can expand the sequence search space and identify candidate molecular variants, whereas top-down proteomics can determine whether sequence variants and PTMs coexist within the same intact protein molecule. Neither approach alone is sufficient to reconstruct the full functional proteoform landscape. A practical maize workflow should therefore use broad bottom-up and phosphoproteomic measurements for discovery, proteogenomics to improve sequence assignment and identify candidate variants, and top-down analysis for targeted resolution of the most biologically informative proteoforms.

A major unresolved question is which recurrent maize stress-responsive proteins contain functionally distinct molecular forms and which of these forms are associated with stress tolerance. Current studies provide evidence for several candidates but rarely establish the relationship between proteoform distribution and physiological phenotype [4,15,56]. Resolving this complexity will require experimental designs capable of linking specific proteoforms to physiological traits and stress-related phenotypes. Overall, proteoforms represent a largely hidden but potentially critical layer of molecular regulation in maize responding to drought and heat stresses. Current proteomic and phosphoproteomic studies have revealed extensive remodeling of protein abundance and phosphorylation networks, yet much of the underlying proteoform diversity remains unexplored. Together, these mechanisms demonstrate that proteoform diversity can arise from multiple molecular processes acting simultaneously, including post-translational modification, proteolytic processing, transcript variation, and genetic variation. In maize stress proteomics, however, the relative contribution of these mechanisms remains incompletely resolved [39,43,53,73].

Among the maize proteins currently representing the strongest candidates for functionally distinct proteoforms are dehydrins, including Rab17/DHN1, HSP26 and other heat shock proteins, antioxidant enzymes, aquaporins, protein kinases, stress-responsive transcription factors, and selected photosynthetic proteins. These candidates differ in the mechanisms expected to generate proteoform diversity: phosphorylation is particularly relevant for dehydrins, kinases, transcription factors, and aquaporins, whereas oxidation and proteolytic processing may be especially important for antioxidant and photosynthetic proteins [92]. The strongest maize-specific evidence currently comes from HSP26, for which distinct isoforms displayed genotype-dependent drought responding, and Rab17/DHN1, for which phosphorylation has functional consequences for intracellular localization and activity. These proteins therefore represent priority candidates for direct proteoform characterization rather than merely recurrent protein-level biomarkers. Despite their biological significance, proteoforms remain largely unresolved in current maize stress research [4]. Most available datasets are generated using bottom-up proteomic workflows, which infer protein identity from peptide measurements but often fail to preserve information regarding the exact combination of modifications present on individual protein molecules [37,75]. The methodological strengths and limitations of the major proteomic approaches with respect to proteoform characterization are summarized in Table 1. Consequently, different proteoforms may be grouped into a single protein identification, obscuring potentially important regulatory mechanisms. This limitation is particularly relevant when studying phosphorylation, where multiple modification sites can generate a large number of distinct phosphoproteoforms from a single protein species.

The synthesis presented here leads to several testable predictions that can guide future maize stress proteomics. First, conserved stress-response pathways should be detectable across genotypes, but their proteoform composition should differ according to stress tolerance. Second, phosphorylation patterns are expected to discriminate drought from heat more effectively than total protein abundance for a subset of regulatory proteins [37,53,75]. Third, combined drought–heat stress should generate non-additive proteoform states rather than simply the sum of the responding observed under individual stresses. Fourth, proteoform-resolved measurements should provide stronger associations with physiological resilience than canonical protein abundance measurements alone. These predictions define a research agenda that extends beyond improving analytical coverage toward experimentally testing the biological role of proteoform diversity.

Many studies remain largely descriptive and continue to focus on protein abundance changes without fully addressing the underlying mechanisms responsible for proteome complexity. Consequently, several important conceptual, methodological, and technological gaps must be addressed to advance the field toward a more comprehensive understanding of stress adaptation [19,93]. One of the most important unresolved questions is whether differences in stress tolerance are associated primarily with changes in protein abundance or with changes in the distribution of molecular forms within recurrent protein families. Most published studies identify proteins as single entities despite the fact that individual proteins frequently exist as heterogeneous populations of molecular variants generated through post-translational modifications, alternative splicing, proteolytic processing, and genetic variation [15,53,92]. As discussed in previous sections, phosphorylation alone can generate multiple functionally distinct proteoforms from a single protein species. However, the biological significance of these proteoforms remains largely unexplored in maize. Figure 1 illustrates how phosphorylation contributes directly to proteoform generation, yet most current datasets lack the resolution required to characterize these molecular species individually. Based on the evidence synthesized above, future proteoform-resolved studies should prioritize proteins for which three criteria converge: recurrent detection across independent stress studies, evidence or strong mechanistic rationale for post-translational or isoform-dependent regulation, and a plausible link to physiological stress tolerance. By these criteria, dehydrins including Rab17/DHN1, HSP26 and other heat shock proteins, antioxidant enzymes, aquaporins, stress-responsive kinases, transcription factors, and selected photosynthetic proteins represent the most informative initial targets. HSP26 and Rab17/DHN1 are particularly strong candidates because maize-specific evidence already demonstrates isoform- or phosphorylation-dependent functional heterogeneity. Kinases and transcription factors should be prioritized for phosphorylation-resolved studies, whereas dehydrins, antioxidant enzymes, and aquaporins provide strong candidates for integrated abundance–PTM–proteoform analyses [74]. Based on the evidence synthesized in this review, we propose a proteoform-centered framework consisting of several major functional modules that could substantially improve the biological interpretation of maize stress proteomics datasets.

A second major gap concerns the limited application of top-down proteomics in plant stress research. The vast majority of maize studies employ bottom-up workflows, which provide excellent proteome coverage but often fail to preserve information regarding the exact combination of modifications present on individual protein molecules. As a result, multiple proteoforms may be collapsed into a single protein identification. If proteoform composition is a determinant of stress resilience, then genotypes displaying contrasting drought tolerance should differ not necessarily in the abundance of canonical proteins, but in the relative distribution of their molecular forms. This prediction can be tested by combining quantitative proteomics with intact-proteoform measurements and physiological phenotyping. Although technical challenges related to protein extraction, separation, and mass spectrometric analysis currently limit its widespread use in plant systems, continued methodological development is expected to expand its applicability in future maize studies [94].

Another important limitation is the relatively narrow focus of most experimental designs. Many investigations examine individual stress factors under controlled laboratory conditions, whereas field-grown maize plants are commonly exposed to combinations of drought, heat, nutrient limitations, and biotic stressors. Drought and heat frequently occur simultaneously during critical developmental stages, producing responding that cannot be accurately predicted from single-stress experiments [68]. Nevertheless, combined-stress phosphoproteomic and proteomic datasets remain scarce [68]. Future research should increasingly incorporate multifactorial experimental designs that better reflect the environmental conditions encountered in agricultural systems. This leads to a testable prediction: if drought and heat interact at the level of shared signaling proteins, combined stress should produce phosphorylation or modification patterns that cannot be reconstructed by simply overlaying the corresponding single-stress datasets. Temporal resolution represents an additional challenge. Stress responding are highly dynamic processes involving rapid signaling events followed by longer-term physiological and metabolic adjustments. However, many published studies rely on single sampling points that provide only a static representation of complex biological processes [75,83]. Early phosphorylation events occurring within minutes of stress perception may differ substantially from proteomic changes observed after prolonged exposure. Time-resolved proteomic and phosphoproteomic investigations are therefore needed to distinguish primary signaling mechanisms from downstream acclimation responding and to identify transient stress-responsive proteoforms.

Current knowledge is also limited by incomplete integration across biological scales. Although transcriptomic, proteomic, and phosphoproteomic datasets are increasingly available, relatively few studies combine these approaches within unified analytical frameworks [75,82]. Figure 2 illustrates how genomic variation, transcript diversity, protein abundance, and post-translational modification collectively contribute to proteoform diversity and stress adaptation. Future research should move beyond isolated omics analyses toward comprehensive multi-omics strategies capable of linking molecular variation to physiological performance. Such approaches may improve mechanistic interpretation and generate testable hypotheses linking molecular variation to stress tolerance phenotypes [37,75]. Proteogenomics represents a particularly promising direction for future maize research. Because maize exhibits extensive genetic diversity, reference protein databases may not adequately capture genotype-specific protein variants and alternative translation products. Integrating genomic sequencing, transcriptomics, and proteomics can improve protein identification and reveal sequence variants, alternative splice products, and other molecular features that may contribute to proteoform diversity. A recent integrated study of two contrasting maize hybrids at the tasseling stage identified 1701 differentially expressed genes but 424 differentially abundant proteins under drought, illustrating the substantial reduction in detectable changes between the transcript and protein levels [95]. However, the direct characterization of the resulting proteoforms will require complementary proteomic approaches capable of resolving intact protein species. Furthermore, combining proteogenomics with phosphoproteomics may provide new insights into how genetic variation influences phosphorylation patterns and signaling network organization [96].

The conceptual framework linking genomic variation, proteoform diversity, and stress adaptation through multi-omics integration is presented in Figure 2 [87].

A major unresolved gap concerns the underrepresentation of reproductive tissues in current proteomic datasets. Most studies focus on leaves and roots because these tissues are readily accessible and highly responsive to environmental stress. However, yield losses in maize are often determined by stress-induced damage occurring in reproductive organs, including tassels, pollen, silks, and developing kernels [97,98]. Proteomic and phosphoproteomic characterization of reproductive tissues may therefore reveal novel molecular mechanisms directly associated with yield stability under drought and heat stresses conditions. The underrepresentation of reproductive tissues is not merely a sampling gap. It may bias the current model of maize stress adaptation toward vegetative survival rather than reproductive resilience. Because yield stability depends strongly on pollen, silk, and kernel responding, proteoform states associated with reproductive tissues may represent a distinct molecular layer of stress tolerance that is poorly captured by leaf- and root-centered studies. Future technological developments are likely to transform maize stress proteomics by increasing analytical depth, spatial resolution, reproducibility, and the ability to resolve low-abundance and functionally distinct proteoforms. Spatial proteomics may reveal tissue- and cell-type-specific stress responding while preserving the spatial organization of maize tissues, which is particularly relevant for heterogeneous organs such as leaves, vascular tissues, roots, and reproductive structures. Single-cell and single-nucleus proteomic approaches could further uncover cellular heterogeneity that is obscured by conventional bulk-tissue analyses, although their application in plant systems remains technically challenging because of the low protein content of individual cells and the complexity of plant tissues. These approaches may be particularly valuable for distinguishing cell-type-specific responding to drought and heat stresses and for linking molecular heterogeneity with physiological adaptation. Ion mobility-enabled proteomics represents another important technological development. The addition of ion mobility separation introduces an additional dimension of peptide separation beyond retention time and mass-to-charge ratio, thereby reducing spectral complexity and improving peptide discrimination. In particular, four-dimensional DIA approaches combining chromatographic separation, mass-to-charge ratio, ion mobility, and intensity offer increased depth and reproducibility and may improve the detection of low-abundance proteins and post-translationally modified peptides. These capabilities are highly relevant to maize stress proteomics, where low-abundance signaling proteins and transient phosphorylation events may otherwise remain undetected. Data-independent acquisition (DIA)-based phosphoproteomics is also emerging as a powerful strategy for comprehensive and reproducible characterization of phosphorylation dynamics. Unlike conventional data-dependent acquisition, DIA systematically samples predefined mass ranges and can provide improved quantitative consistency across large sample series. This is particularly important for stress experiments involving multiple genotypes, tissues, time points, or combined drought and heat treatments [68]. The integration of DIA phosphoproteomics with ion mobility separation may therefore provide a powerful framework for capturing both abundant and low-abundance phosphopeptides and for resolving dynamic phosphorylation changes associated with stress signaling. Advances in artificial intelligence and machine learning are expected to further enhance these technological developments. AI-assisted approaches can support peptide and protein identification, spectral interpretation, protein function prediction, annotation of poorly characterized proteins, and integration of proteomic, phosphoproteomic, genomic, and transcriptomic datasets. For maize, such approaches may be particularly valuable because extensive genetic variation and incomplete functional annotation can complicate protein identification and biological interpretation. AI-based annotation combined with proteogenomics could therefore help prioritize previously uncharacterized proteins and candidate proteoforms associated with drought and heat resilience [99]. Machine learning and artificial intelligence are increasingly becoming integral to proteomic data interpretation, extending beyond conventional statistical analysis toward peptide-spectrum matching, protein identification, functional annotation, spectral prediction, and multi-omics integration. In maize, AI-assisted annotation may be particularly useful for proteins that are poorly characterized or absent from standard reference annotations because of genotype-specific sequence variation, alternative splicing, or incomplete functional characterization. Integration of AI-based protein annotation with proteogenomic and phosphoproteomic data could therefore facilitate the identification and prioritization of previously unrecognized stress-responsive proteins and proteoforms. However, AI-derived annotations should be treated as hypothesis-generating and require experimental validation, particularly when assigning biological functions to novel maize proteins. Figure 2 highlights the importance of such integrative frameworks for connecting molecular variation with phenotypic outcomes [87]. Beyond methodological advances, future research should place greater emphasis on the functional validation of candidate proteins and proteoforms. Many stress-associated proteins have been repeatedly identified across independent studies, yet their causal contribution to stress tolerance often remains uncertain. Combining quantitative proteomics with genome editing technologies, transgenic approaches, and targeted biochemical analyses will be essential for establishing direct links between specific proteoforms and adaptive phenotypes [75].

Significant opportunities remain for advancing our understanding of maize adaptation to drought and heat stresses. Addressing current limitations through proteoform-resolved approaches [37], DIA and ion mobility-enabled proteomics, spatial and single-cell technologies [75], integrated multi-omics, proteogenomics [82], AI-assisted data interpretation [99], and functional validation will be essential for resolving the hidden complexity of the maize stress proteome. As illustrated by the conceptual frameworks presented in Figure 1 and Figure 2, future progress will depend on linking phosphorylation-dependent signaling, proteoform diversity, and genotype-specific molecular variation within unified models of stress adaptation. Such efforts may ultimately facilitate the development of climate-resilient maize cultivars capable of sustaining productivity under increasingly challenging environmental conditions [43,94]. The increasing availability of publicly deposited maize proteomic datasets, including recent drought datasets comparing contrasting genotypes, also creates opportunities for cross-study reanalysis and validation of proteoform-related hypotheses [100].

## 8. Conclusions

The cross-study evidence reviewed here indicates that maize responses to drought and heat converge at the level of several major functional processes, including photosynthetic adjustment, redox control, proteostasis, metabolism, and stress signaling, while the abundance and regulation of individual proteins can vary substantially among genotypes, tissues, developmental stages, and stress regimes. This apparent variability should therefore not be interpreted simply as a lack of biological consistency. Differences in experimental context and analytical resolution can produce distinct protein-level readouts of related adaptive processes, emphasizing the importance of evaluating stress responding at the level of functional modules rather than relying exclusively on individual protein abundance. A second conclusion is that protein abundance alone provides an incomplete representation of stress-responsive molecular states. Evidence from maize demonstrates that distinct molecular forms of the same protein can respond differently to environmental conditions, as illustrated by genotype-dependent HSP26 forms and phosphorylation-dependent states of the dehydrin Rab17/DHN1. Moreover, integrated proteomic and phosphoproteomic measurements show that changes in phosphorylation can occur independently of changes in total protein abundance. These observations indicate that biologically meaningful differences may be obscured when distinct molecular species are collapsed into a single protein-level assignment. The practical implication is not that conventional bottom-up proteomics should be replaced, but that it should be complemented by approaches capable of resolving molecular heterogeneity. Quantitative proteomics remains essential for defining the broad landscape of stress-responsive proteins, whereas phosphoproteomics provides access to rapid regulatory changes, and proteogenomics can improve the identification of genotype-specific sequence variants and alternative protein products. Top-down and other intact-protein strategies are particularly important when the biological question concerns the composition and functional properties of individual proteoforms, although their current analytical limitations require targeted rather than indiscriminate application. The most informative future studies will therefore combine these approaches with physiological and genetic validation rather than treating any single proteomic platform as sufficient on its own. This integrated strategy should be applied preferentially to proteins for which recurrent stress responsiveness is accompanied by a plausible capacity for functional proteoform diversification, including dehydrins, heat shock proteins, signaling and transcriptional regulators, aquaporins, antioxidant enzymes, and selected photosynthetic proteins. Priority should also be given to reproductive tissues and contrasting genotypes, where proteoform composition may provide a more direct link between molecular regulation and yield stability than measurements performed predominantly in vegetative tissues. Such targeted studies would allow the field to move from cataloguing stress-responsive proteins toward testing whether specific molecular states actually distinguish stress damage from successful acclimation. Overall, current maize stress proteomics has generated an extensive and valuable foundation of protein- and phosphoprotein-level data, but the next conceptual advance will require resolving how genetic variation, post-translational modification, proteolytic processing, and alternative protein products combine to generate functionally distinct molecular states. The central challenge is therefore no longer simply to determine which proteins respond to drought or heat but to identify which proteoforms are present, how their abundance and modification states change under stress, and which of these states are causally associated with physiological resilience. Addressing this challenge through integrated proteomics, phosphoproteomics, proteogenomics, and targeted proteoform-resolved analyses could provide a more mechanistic basis for understanding maize stress adaptation and for identifying molecular targets relevant to climate-resilient crop improvement.

## Figures and Tables

**Table 1:** Comparison of major proteomic approaches used in maize stress research with emphasis on proteoform characterization.

Approach	Primary Analytical Target	Major Strengths	Major Limitations	Ability to Resolve Intact Proteoforms	Representative Reference
2-DE	Intact protein species separated by pI and molecular mass	Direct visualization of protein species; detection of protein isoforms, PTM-dependent shifts and proteolytic variants; strong biological interpretability; excellent for comparative proteoform analysis	Lower proteome coverage than shotgun workflows; reduced performance for some highly hydrophobic proteins; labor-intensive	Moderate–High for protein-species separation; limited for definitive proteoform characterization	[29,30,31,32]
2D-DIGE	Intact protein species with fluorescent multiplex quantification	All advantages of 2-DE plus improved quantitative accuracy, reproducibility and direct comparison of samples within a single gel	Similar constraints to 2-DE; lower throughput than LC-MS/MS workflows	Moderate–High for protein-species separation; limited for definitive proteoform characterization	[30,31,32]
Bottom-up LC-MS/MS	Peptides generated by enzymatic digestion	High proteome coverage; high sensitivity; suitable for large-scale discovery studies	Protein identities inferred from peptides; relationships among coexisting proteoforms often lost; PTM combinations cannot usually be assigned to individual protein molecules	Low	[24,28,33]
Quantitative LC-MS/MS (LFQ, TMT, iTRAQ)	Relative peptide abundance across samples	Accurate quantitative comparisons; multiplexing capability; high throughput	Same proteoform-related limitations as bottom-up proteomics; ratio compression (TMT/iTRAQ); workflow-dependent biases	Low	[24,28,33]
Phosphoproteomics	Phosphopeptides and phosphorylation sites	Direct characterization of phosphorylation networks; identification of stress-responsive phosphosites; insight into signaling pathways	Depends on enrichment efficiency, phosphosite localization and phosphopeptide detectability; usually does not resolve complete phosphoproteoforms	Low–Moderate	[19,24]
MS-intensive Top-Down Proteomics (MSi-TDP)	Intact proteins and proteoforms	Direct characterization of proteoforms; simultaneous assessment of sequence variants and PTMs on individual protein molecules	Technically demanding; limited throughput; challenges with large proteins, complex mixtures and plant proteomes; currently biased toward lower-molecular-weight proteoforms	High/Direct	[33,34,35,36]
Proteogenomics	Genome-informed protein identification	Enables detection of sequence variants, alternative splice products and previously unannotated coding regions; improves protein annotation	Does not directly resolve intact proteoforms; typically relies on bottom-up peptide evidence; computationally intensive	Indirect	[36]

Proteoform resolution refers to the ability of a methodology to distinguish and characterize distinct molecular forms originating from a single gene product. High proteome coverage does not necessarily imply high proteoform resolution, and different analytical approaches provide complementary views of proteome organization.

**Table 2:** Frequently reported proteins exhibiting altered abundance in maize tissues exposed to drought stress. The table summarizes major functional categories identified across proteomic studies and highlights potential sources of proteoform diversity arising from post-translational modifications (PTMs), proteolytic processing, or protein isoforms. Because most published studies employ bottom-up proteomic workflows, the reported changes generally refer to protein groups or inferred protein species rather than directly characterized proteoforms [40,41,43,44,45,46,47,48,49,50].

Protein/Protein Family	Typical Abundance	Biological Function	Tissue(s) Most Frequently Reported	Potential Proteoform Relevance
Ribulose-1,5-bisphosphate carboxylase/oxygenase (Rubisco) large and small subunits	Mostly decreased	Carbon fixation and photosynthesis	Leaves	Susceptible to oxidation, proteolytic cleavage, and phosphorylation, generating multiple stress-associated proteoforms [40,41,43,47]
Oxygen-evolving enhancer proteins (OEE1, OEE2)	Decreased	Stabilization of photosystem II	Leaves	Phosphorylation-dependent forms may influence photosynthetic adaptation [43]
Chlorophyll a/b-binding proteins (LHC proteins)	Mostly decreased	Light harvesting and energy transfer	Leaves	Multiple phosphorylated proteoforms involved in photoprotection [41]
ATP synthase subunits	Variable	ATP production	Leaves, roots	PTMs may regulate energy metabolism during dehydration [43,48]
Fructose-bisphosphate aldolase	Variable	Glycolysis and Calvin cycle	Leaves, roots	Multiple isoforms and phosphorylation sites reported [40,41]
Glyceraldehyde-3-phosphate dehydrogenase (GAPDH)	Mostly decreased	Energy metabolism and redox signaling	Leaves, roots	Known to undergo oxidation, nitrosylation, and phosphorylation [40,43]
Enolase	Increased	Glycolysis	Leaves, roots	Stress-induced PTMs may alter enzymatic activity [41]
Malate dehydrogenase	Increased	TCA cycle and energy metabolism	Roots, leaves	Multiple mitochondrial and cytosolic proteoforms occur [40,43]
Superoxide dismutase (SOD)	Increased	Detoxification of reactive oxygen species	Leaves, roots	Activity influenced by oxidation and phosphorylation [40,41,43]
Catalase (CAT)	Increased	Hydrogen peroxide detoxification	Leaves	Multiple modified forms associated with oxidative stress adaptation [40,43]
Ascorbate peroxidase (APX)	Increased	Antioxidant defense	Leaves, roots	Redox-dependent proteoforms reported [40,43]
Glutathione S-transferase (GST)	Increased	Detoxification and stress defense	Leaves, roots	Large family with numerous isoforms and PTMs [41]
Peroxiredoxins	Increased	ROS scavenging	Leaves	Oxidation state generates functionally distinct proteoforms [40,43]
Late embryogenesis abundant proteins (LEA)	Increased	Cellular protection during dehydration	Leaves, roots, embryos	Extensive phosphorylation reported in several plant species [40]
Dehydrins	Increased	Membrane and protein stabilization	Leaves, roots	Phosphorylation affects localization and function [40]
Heat shock proteins (HSP70, HSP90, small HSPs)	Increased	Protein folding and proteostasis	Leaves, roots, reproductive tissues	Multiple stress-dependent proteoforms generated through PTMs [40,43]
Chaperonins	Increased	Protein quality control	Leaves	Potential phosphorylation-dependent functional variants [40]
14-3-3 proteins	Increased	Signal transduction and protein interaction	Leaves, roots	Function determined by phosphorylation-dependent interactions [43]
Calcium-binding proteins	Increased	Stress signaling	Roots, leaves	PTMs may modulate signaling activity [43,44]
Protein kinases (including SnRK-related proteins)	Altered	Stress signaling and ABA responding	Leaves, roots	Frequently detected as phosphoproteins generating multiple proteoforms [43,44]
Proteasome subunits	Increased	Degradation of damaged proteins	Leaves, roots	Ubiquitination-related proteoform dynamics [40]
Aquaporins	Variable	Water transport	Roots, leaves	Phosphorylation regulates channel activity [43,49]
Late-stage ABA-responsive proteins	Increased	Drought adaptation	Leaves, roots	Potentially represented by multiple phosphorylated proteoforms [43,50]

**Table 3:** Frequently reported proteins exhibiting altered abundance in maize tissues exposed to heat stress. The table summarizes major protein groups identified across proteomic studies and highlights potential mechanisms contributing to proteoform diversity, including phosphorylation, oxidation, acetylation, ubiquitination, and proteolytic processing. Because most available datasets originate from bottom-up proteomic workflows, direct characterization of individual proteoforms remains limited.

Protein/Protein Family	Typical Change in Abundance Under Heat Stress	Biological Function	Tissue(s) Most Frequently Reported	Potential Proteoform Relevance
Heat shock proteins (sHSPs)	Increased abundance	Prevention of protein aggregation and stabilization of unfolded proteins	Leaves, pollen, kernels	Multiple phosphorylated and oligomeric proteoforms reported in plants [33,56]
HSP70 family	Increased abundance	Protein folding and refolding	Leaves, roots, reproductive tissues	Phosphorylation and acetylation may alter chaperone activity [33,56]
HSP90 family	Increased abundance	Stabilization of signaling proteins and protein complexes	Leaves, reproductive tissues	PTMs influence client-protein interactions [33]
HSP100/Clp proteins	Increased abundance	Reactivation of aggregated proteins	Leaves	Distinct proteoforms generated by phosphorylation and proteolytic processing [33]
Chaperonins (CPN60 family)	Increased abundance	Protein quality control in chloroplasts and mitochondria	Leaves	Potential phosphorylation-dependent variants [33]
Rubisco activase	Frequently decreased abundance	Maintenance of Rubisco catalytic activity	Leaves	Heat-sensitive proteoforms with distinct thermal stability [60]
Rubisco large and small subunits	Decreased abundance	Carbon fixation	Leaves	Oxidation, proteolytic cleavage, and phosphorylation contribute to proteoform diversity [33,56,60]
Oxygen-evolving enhancer proteins (OEE1/OEE2)	Decreased abundance	Stabilization of photosystem II	Leaves	PTMs may affect photosynthetic resilience [56,60]
Chlorophyll a/b-binding proteins	Decreased abundance	Light harvesting	Leaves	Phosphorylation-related proteoforms regulate energy distribution [33,60]
ATP synthase subunits	Altered abundance	ATP production	Leaves, roots	Multiple PTMs associated with energy regulation [56,60]
Ferredoxin-NADP reductase	Decreased abundance	Photosynthetic electron transport	Leaves	Potential oxidative proteoforms under thermal stress [56,60]
Fructose-bisphosphate aldolase	Increased or decreased abundance	Glycolysis and carbon metabolism	Leaves, roots	Known phosphorylation sites reported in cereals [56,60]
Glyceraldehyde-3-phosphate dehydrogenase (GAPDH)	Increased abundance	Energy metabolism and redox regulation	Leaves	Oxidation- and phosphorylation-dependent proteoforms [33,60]
Enolase	Increased abundance	Glycolysis	Leaves, roots	PTMs may modify catalytic efficiency [56,60]
Malate dehydrogenase	Altered abundance	TCA cycle and respiration	Leaves, roots	Distinct organellar proteoforms occur [33,60]
Superoxide dismutase (SOD)	Increased abundance	ROS detoxification	Leaves, roots	Oxidative and phosphorylated proteoforms described [33,56,60]
Catalase (CAT)	Increased abundance	Hydrogen peroxide scavenging	Leaves	Activity influenced by PTMs and oxidation status [33,60]
Ascorbate peroxidase (APX)	Increased abundance	Antioxidant defense	Leaves	Multiple stress-associated proteoforms possible [56,60]
Glutathione S-transferases (GSTs)	Increased abundance	Detoxification and oxidative stress protection	Leaves, roots	Large family with numerous isoforms and PTMs [56,60]
Peroxiredoxins	Increased abundance	Redox homeostasis	Leaves	Functional diversity linked to oxidation state [33,60]
14-3-3 proteins	Increased abundance	Signal transduction and protein interaction	Leaves, roots	Phosphorylation-dependent interaction networks [43,60]
Calcium-binding proteins	Increased abundance	Stress signaling	Leaves, reproductive tissues	PTMs may regulate signaling efficiency [43,60]
Protein kinases (MAPKs, CDPKs)	Altered abundance	Stress signal transduction	Leaves, roots	Frequently detected as phosphoproteins generating multiple proteoforms [43,60]
Ubiquitin-conjugating enzymes	Increased abundance	Protein degradation and turnover	Leaves	Central components of proteoform regulation [43,60]
Proteasome subunits	Increased abundance	Removal of damaged proteins	Leaves, roots	Ubiquitination-related proteoform dynamics [60]
Elongation factors (EF-Tu, EF-1α)	Altered abundance	Protein synthesis	Leaves	Heat-induced PTMs may influence translational efficiency [60]
Actin and tubulin proteins	Altered abundance	Cytoskeleton organization	Pollen, anthers, leaves	Multiple PTMs affect polymerization dynamics [33,60]
LEA proteins	Increased abundance	Cellular protection under stress	Leaves, reproductive tissues	Frequently phosphorylated in stress conditions [56,60]
Dehydrins	Increased abundance	Membrane and protein stabilization	Leaves, roots	Phosphorylation influences localization and activity [33]

Notably, more than half of the protein families repeatedly identified in maize heat stress studies are known targets of post-translational modifications. This observation suggests that heat-induced adaptation may depend not only on changes in protein abundance but also on the generation of functionally distinct proteoforms that remain largely unresolved in current maize proteomic datasets.

## Data Availability

No new data were created or analyzed in this study. Data sharing is not applicable to this article.

## Abbreviations

The following abbreviations are used in this manuscript:

PTM	Post-translational modification
MS	Mass spectrometry
LC-MS/MS	Liquid chromatography–tandem mass spectrometry
2-DE	Two-dimensional gel electrophoresis
LFQ	Label-free quantification
TMT	Tandem mass tag
iTRAQ	isobaric tags for relative and absolute quantification
IMAC	Immobilized metal affinity chromatography
TiO_2_	Titanium dioxide
OEE	Oxygen-evolving enhancer
GAPDH	Glyceralde-hyde-3-phosphate dehydrogenase
SOD	Superoxide dismutase
CAT	Catalase
APX	Ascorbate peroxidase
GST	Glutathione S-transferase
ROS	Reactive oxygen species
LEA	Late embryogenesis abundant
HSP	Heat shock protein
ABA	Abscisic acid
CDPK	Calcium-dependent protein kinase
CIPK	CBL-interacting protein kinase
RLK	Receptor-like kinase
FNR	Ferredoxin-NADP reductase
SnRK2	SNF1-related protein kinase 2
MAPK	Mitogen-activated protein kinase
MAPKs	MAP kinases
MAPKKs	MAP kinase kinases
MAPKKKs	MAP kinase kinase kinases
